# Neuronal Avalanches Across the Rat Somatosensory Barrel Cortex and the Effect of Single Whisker Stimulation

**DOI:** 10.3389/fnsys.2021.709677

**Published:** 2021-08-30

**Authors:** Benedetta Mariani, Giorgio Nicoletti, Marta Bisio, Marta Maschietto, Roberto Oboe, Alessandro Leparulo, Samir Suweis, Stefano Vassanelli

**Affiliations:** ^1^Laboratory of Interdisciplinary Physics, Department of Physics and Astronomy, University of Padova, Padova, Italy; ^2^Padova Neuroscience Center, University of Padova, Padova, Italy; ^3^Department of Biomedical Science, University of Padova, Padova, Italy; ^4^Department of Management and Engineering, University of Padova, Padova, Italy

**Keywords:** brain criticality, avalanches, local field potential (LFP), multi-unit activities, evoked potential, somatotopy, sensory coding

## Abstract

Since its first experimental signatures, the so called “critical brain hypothesis” has been extensively studied. Yet, its actual foundations remain elusive. According to a widely accepted teleological reasoning, the brain would be poised to a critical state to optimize the mapping of the noisy and ever changing real-world inputs, thus suggesting that primary sensory cortical areas should be critical. We investigated whether a single barrel column of the somatosensory cortex of the anesthetized rat displays a critical behavior. Neuronal avalanches were recorded across all cortical layers in terms of both multi-unit activities and population local field potentials, and their behavior during spontaneous activity compared to the one evoked by a controlled single whisker deflection. By applying a maximum likelihood statistical method based on timeseries undersampling to fit the avalanches distributions, we show that neuronal avalanches are power law distributed for both multi-unit activities and local field potentials during spontaneous activity, with exponents that are spread along a scaling line. Instead, after the tactile stimulus, activity switches to a transient across-layers synchronization mode that appears to dominate the cortical representation of the single sensory input.

## 1. Introduction

The cortex operates in a state of restless activity, whose meaning and functionality are not yet understood. The critical brain hypothesis suggests that this is the result of the brain operating in the vicinity of the critical point of a phase transition, leading to a rich and variable dynamics at rest. In general, it has been argued that criticality provides biological systems with an optimal balance between robustness against perturbations and the flexibility to adapt to changing conditions. In the case of the brain, this would confer optimal computational capabilities [e.g., by optimizing the correlation length and the dynamic range, leading to the existence of large dynamical repertoires accompanied by maximal transmission and storage of information (Kinouchi and Copelli, 2006; Shew et al., 2009; Shew and Plenz, 2013; Hidalgo et al., 2014; Muñoz, 2018)]. In this context, Hidalgo et al. (2014) have shown that complex adaptive systems that have to cope with a great variety of stimuli are much more efficient when operating in the vicinity of a critical point, and thus they benefit from dynamically tuning themselves to that point.

By analyzing LFPs of cortical neurons in culture, the seminal work of Beggs and Plenz (2003) provided the first evidence of power law distributed neuronal avalanches, i.e., cascades of activity interspersed by periods of quiescence typical of critical systems. In particular, the exponents of these power laws were remarkably close to the ones of a critical branching process, hence suggesting a scenario in which neuronal networks are characterized by a marginal propagation of the activity at the critical point between an active and an absorbing phase. Since then, such power laws have been observed repeatedly in different experimental settings (Mazzoni et al., 2007; Gireesh and Plenz, 2008; Pasquale et al., 2008; Petermann et al., 2009; Hahn et al., 2010; Yu et al., 2011), thus strengthening the critical brain hypothesis. Despite that, ambiguities, and open questions remain.

A first problem concerns the experimental definition of avalanche. In order to estimate avalanches, one needs to define discrete events. While neuronal spikes and multi-unit activities (MUAs) are events by nature, the conversion of coarse-sampled brain signals such as LFPs into a discrete form (e.g., by simply applying a threshold) is more ambiguous and difficult to interpret in terms of neural correlates. As the relation between events and actual underlying neural activity becomes more uncertain, the definition of neural avalanches becomes fuzzy (Touboul and Destexhe, 2010, 2017; Dalla Porta and Copelli, 2019; Villegas et al., 2019).

An additional limiting factor is spatial sampling of experimental recordings. Studies using coarse-sampled activity like LFPs typically yielded power-law distributions both *in vivo* and *in vitro*, but several experiments relying on spikes in awake animals did not (Ribeiro et al., 2010; Dehghani et al., 2012; Priesemann et al., 2014). One possible cause is insufficient spatial sampling of recording in awake conditions. Avalanches are a population phenomenon, but spikes are sparsely recorded in these experiments and reflect a subpopulation of neurons, in fact missing a consistent fraction of the real activity (Ribeiro et al., 2010). On the other hand, LFPs are average and composite signals that reflect neuronal populations but are difficult to interpret in terms of single neurons activity (Einevoll et al., 2013; Wilting and Priesemann, 2019; Neto et al., 2020). This makes the event-extraction process less straightforward and threshold dependent (Touboul and Destexhe, 2010), even though nLPFs are known to correlate with synchronous spiking activity (Beggs and Plenz, 2003; Petermann et al., 2009; Yu et al., 2011). For all these reasons, it is informative to include spikes, MUAs and LFPs when measuring neural avalanches (see for example Hahn et al., 2010).

Up to now, most of the work on neuronal avalanches has focused on spontaneous activity, while much remains to be understood about their behavior after perturbations caused by incoming inputs. Investigating the response to sensory stimuli in primary cortical areas is a clear-cut strategy to address this point. First, these brain regions can be expected to benefit from operating around a critical point to encode the sensory stimuli themselves. Moreover, sensory inputs, which are under direct experimental control, propagate to cortical networks across a limited number of well characterized processing stages (contrary to, e.g., associative or motor areas). Avalanches were studied in the turtle visual cortex *ex-vivo* while the retina was exposed to a movie acting as a continuous visual stimulus (Shew et al., 2015; Clawson et al., 2017). Based on neural avalanches extracted from LFPs, results suggested that the cortical network self-adapts to a critical state after a short period from the stimulus. However, in these experiments, LFPs were sparsely measured and reflected populations of neurons scattered across the visual processing cascade and downstream to important processing structures including the retina. Work on the primary auditory cortex hinted, instead, at a critical behavior both in resting and post-stimulus conditions (Bowen et al., 2019). Noteworthy, measurements were confined to either layers 2/3 or 4 and limited by the slow dynamics of calcium imaging to obtain an indirect estimate of neural activity.

In this work we contribute to verify the critical brain hypothesis with a systematic study of neuronal avalanches in the rat barrel cortex (the region of the rat primary somatosensory cortex that encodes tactile sensory inputs from the whiskers). We run our measurements across cortical layers in single barrel columns of the rat anesthetized with tiletamine. This common preparation for electrophysiology (Sorrenti et al., 2021) displays rich cortical spontaneous activity, including UP and DOWN states and oscillations that have been linked to avalanches and criticality (Scarpetta and de Candia, 2014; di Santo et al., 2018). Given the current challenges to test the critical brain hypothesis and the various nature of recorded signals (Ribeiro et al., 2010; Dehghani et al., 2012; Priesemann et al., 2014), we explored activity across a wide frequency range, covering spikes, MUAs and LFPs (i.e., up to 3,000 Hz). In particular, we investigated both spontaneous activity and the evoked activity that followed a single whisker deflection and analyzed neural avalanches through a protocol based on state-of-the-art maximum likelihood statistical method (Gerlach and Altmann, 2019).

## 2. Materials and Methods

### 2.1. Electrophysiological Recordings and Surgical Procedures

**Figure 1:**
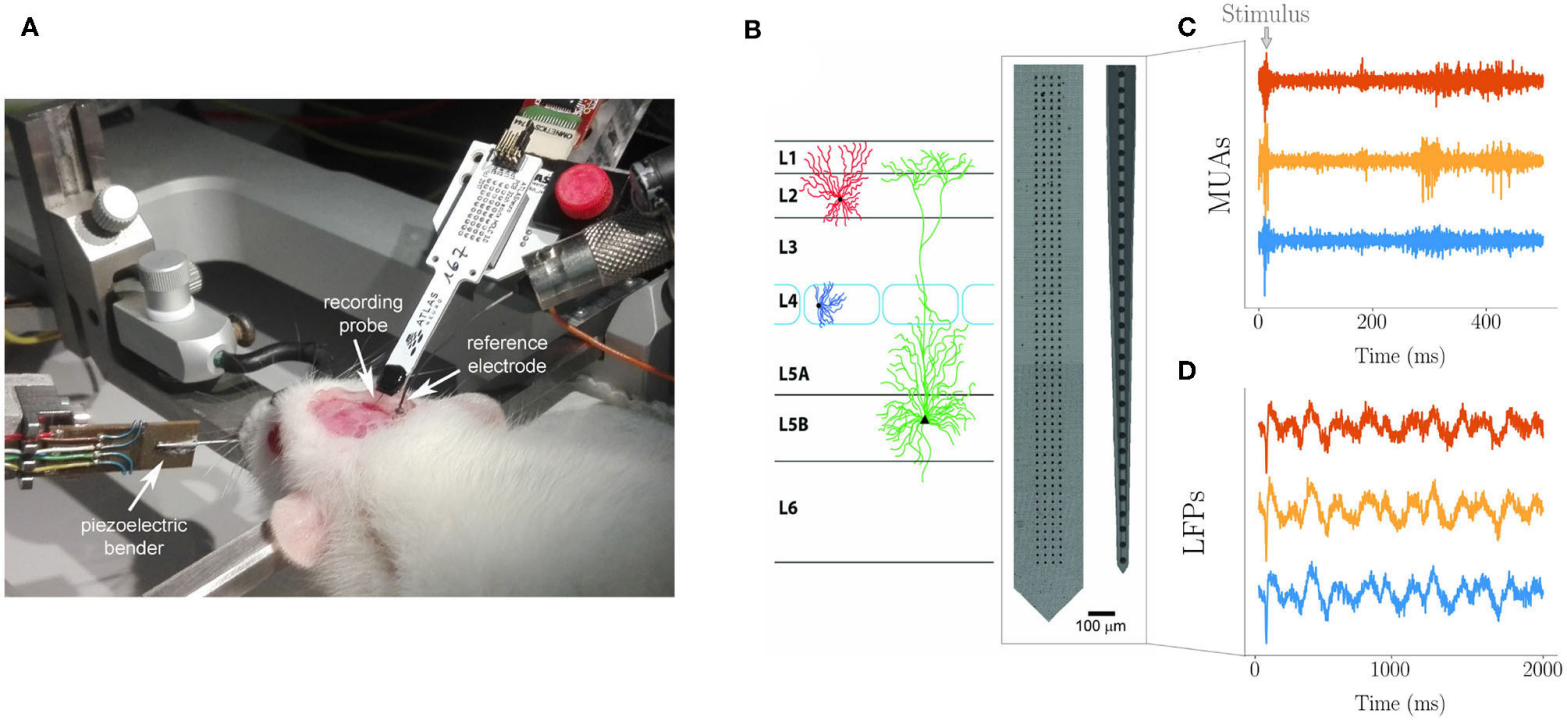
**(A)** Experimental setting for recording with the anesthetized rat immobilized on the stereotaxic apparatus. The Atlas probe for spikes and MUAs recording is inserted in the barrel cortex through the dedicated cranial window. The cortical surface is bathed by Krebs' solution that is grounded through the immersed Ag/AgCl reference electrode. The piezoelectric bender with cannula used to control single whisker deflection is visible on the left. An identical arrangement, but with the custom high-density probe, was adopted for LFPs recording. **(B)** Custom 2D 64 × 4 array used for LFPs and commercial 1D array used for MUAs spanning across cortical layers as during recording (schematic drawing). The actual number of electrodes spanning the barrel cortex was 220 for LFPs (organized in a 55 × 4 matrix form) and 27 for MUAs, with the other electrodes of the arrays remaining above the cortical surface bathed by the grounded electrolyte. Examples of MUAs **(C)** and LFPs **(D)** traces (from three representative channels each).

**Extracellular spikes and MUAs and LFPs recordings**. Spikes and MUAs were recorded using a neural probe with a linear array of 32 Iridium Oxide (IrOx) microelectrodes with 65 μm inter-electrode distance and 2015 μm array length (E32+R-65-S1-L6 NT; Atlas Neuroengineering) (Figure 1). Raw signals were acquired by an Open Ephys Acquisition Board (OEps Tech, Lisbon, Portugal) with a 32-channels head stage (RHD2000, Intan Technologies) and an SPI cable at 25 KHz sampling frequency and band-pass filtered (300–3,000 Hz). Single unit activity (spikes) represented only about one percent of the activity recorded which was, therefore, mostly represented by MUAs. The maximum number of neurons contributing to a MUA is of a few tens per microelectrode as it can be estimated assuming a spherical volume of tissue centered around the recording site, an average density of neurons in the rat barrel cortex ~ 55,000/mm3 from Ren et al., 1992) and and approximate distance of one hundred micrometers at which the amplitude of an extracellular action potential is above the background noise (~ 10 microvolts RMS in the 0.1–3 kHz bandwidth). LFPs were recorded at high density using a CMOS based neural probe with an array of 256 microelectrodes (7.4 μm in diameter size and organized in four vertical columns and sixty-four horizontal rows) (Schroder et al., 2015) with 32 μm inter-electrode distance along both the horizontal and vertical axis and 2023.4 μm array length (Figure 1). Raw multiplexed signals were acquired through a NI PXIe-6358 (National Instruments) board (sampling frequency 1.25 MS/s at 16 bit) and demultiplexed using a custom-made LabVIEW software. The resulting whole-array LFP signal was sampled at 976.56 Hz and band-pass filtered (2–300 Hz). Once inserted in the barrel column, both arrays were spanning across all the six cortical layers (from 0 to 1,800 μm).

**Surgical implantation and single whisker stimulation**. Wistar rats were maintained under standard environmental conditions in the animal research facility of the Department of Biomedical Sciences - University of Padova. All the procedures were approved by the local Animal Care Committee (O.P.B.A.) and the Italian Ministry of Health (authorization number 522/2018-PR). Rats of both sexes, aged 36–50 days (P36-P50) and weighting between 150 and 230 g, were anesthetized with an intra-peritoneal induction mixture of tiletamine-xylazine (2 mg and 1.4 g/100 g body weight, respectively), followed by additional doses (0.5 mg and 0.5 g/100 g body weight) every hour. The anesthesia level was constantly monitored by testing the absence of eye and hind-limb reflexes and whiskers' spontaneous movements. Before starting with surgery, the rat was fixed on a stereotaxic apparatus by teeth and ear bars. The body temperature was monitored continuously with a rectal probe and maintained at 37°C by a heating pad. The skull was exposed through an anterior-posterior opening of the skin in the center of the head and a window was drilled over the right somatosensory barrel cortex at stereotaxic coordinates from −1 to −4 AP, from +4 to +8 ML referred to bregma (Swanson, 2003). A slit in the meninges was made with dedicated fine forceps at coordinates −2.5 AP, +6 ML for the subsequent insertion of the recording probe, and the brain was constantly bathed in Krebs' solution (in mM: NaCl 120, KCl 1.99, NaHCO_3_ 25.56, KH_2_PO_4_ 136.09, CaCl_2_ 2, MgSO_4_ 1.2, glucose 11).

The recording probe was fixed to a dedicated holder connected to a Patchstar micromanipulator (Scientifica Ltd, East Sussex, UK), which was used for inserting the probe into the cortex orthogonal to the cortical surface. The depth was set at 0 μm when the electrode proximal to the chip tip touched the cortical surface. An Ag/AgCl electrode bathed in Krebs' solution in proximity of the probe was used as reference.

Contralateral whiskers were trimmed at around 10 mm from the mystacial pad. To control deflection, single whiskers were inserted for 8 mm inside a cannula glued to a piezoelectric bender with integrated strain gauges (P-871.122; Physik Instrumente (PI) GmbH & Co. KG) and driven by a custom-made digital closed-loop control system. Each stimulus, delivered by a waveform generator (Agilent 33250A 80 MHz, Agilent Technologies Inc., Colorado, USA), was consisting of a voltage pulse of 5 ms duration and 100 μs rise/fall time applied to the piezoelectric bender. The principal (maximally responding) whisker identified on the basis of the amplitude of evoked LFP responses was selected for the recording session.

### 2.2. Avalanches Analysis

**Figure 2:**
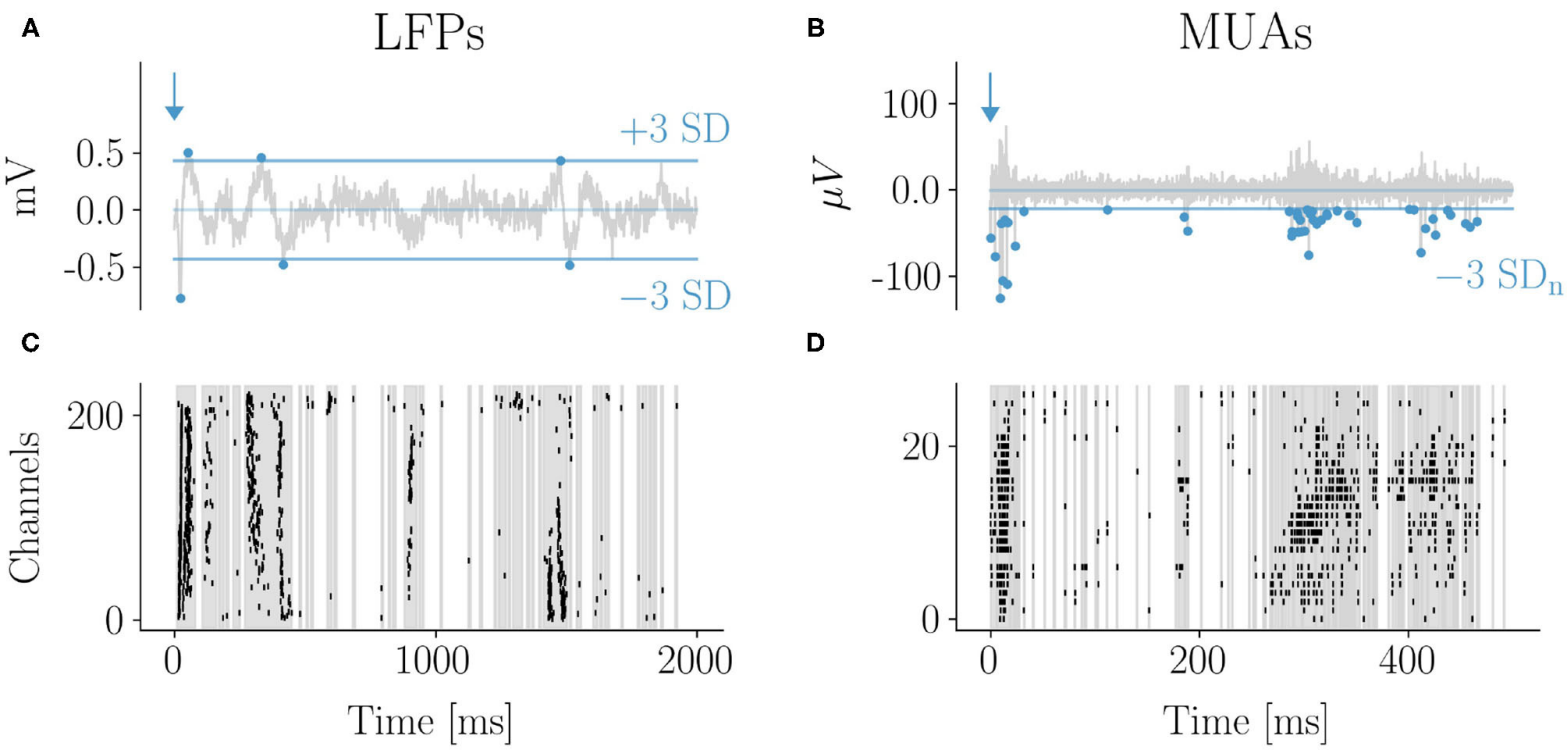
Evoked response by whisker stimulation: LFPs and MUAs. Example of LFP **(A)** and spike **(B)** traces with the 3 standard deviations (SD) thresholds used for events detection (indicated by dots). The corresponding raster plots are shown in **(C,D)**. The 2D array in **(C)** was rearranged in one dimension (groups of four sensors in a row are reported consecutively along the vertical axis). The early response to the stimulus occurred within few tens of milliseconds and was characterized by a high degree of events synchronization across channels both for LFPs and MUAs. The typical LFP evoked response consisted in a first negative peak followed by a slower positive wave. Both were detected as events which is reflected in the raster plot. After the early response large and frequent avalanches typically followed, taking the form of oscillations of synchronous events across channels fading after a few hundreds of milliseconds from the stimulus. Correspondingly, epochs of high frequency firing (bursts) were observed in the MUAs domain **(C,D)**.

MUAs and high-density LFPs recordings were performed separately in four and five rats, respectively. The minimum time interval between consecutive whisker stimuli was set to 2 s to avoid receptors and central adaptation phenomena. Accordingly, 2 s of recording after the stimuli were excluded from the analysis of *basal* activity. Due to the different duration of the stimulus-evoked avalanches in the LFPs and MUAs domains (Figure 2), *post-stimulus* intervals were set for analysis at 2 s or 500 ms for LFPs and MUAs, respectively. In total, 2 min long recordings of LFPs basal activity and 5 min long recordings of MUAs basal activity were analyzed for each rat. Forty stimulations of the whisker were considered for each rat in the analysis of LFPs evoked activity, while in MUAs data the recordings of each rat included at least 60 stimulations of the whisker.

For the detection of LFP events, the standard deviation (SD) and the mean of the signal was computed for each channel. In order to distinguish real events from noise, a three SD threshold was chosen basing on the distribution of the signal amplitudes which significantly deviated from a Gaussian best fit above that threshold (see Supplementary Figure 1). Both negative and positive LFPs (i.e., nLPFs adn pLFPs, respectively) were considered as events in accordance with previous work (Shew et al., 2015; Clawson et al., 2017). Within our specific experimental settings, one reason is that across the depth of the cortex there are polarity changes of the LFP signal because of compensatory capacitive ionic currents particularly along dendrites of pyramidal cells (Buzsaki et al., 2012; Einevoll et al., 2013). Since in our experiments electrodes span multiple cortical layers, both nLFPs and pLFPs were found and detected. Moreover, alternatively, pLFPs can be related to activation of populations of inhibitory neurons leading to inhibitory outward postsynaptic currents, which also justifies their inclusion in the events count. For detection, each deflection was considered terminated only after it crossed the mean of the signal. For completeness, we report analyses on nLFPs only, and also considering separately the superficial layers of the cortex and the bottom layers (Supplementary Figures 8–13).

For extracellular spikes and MUAs the detection threshold was set at three SD of the noise (Quiroga et al., 2004). In fact, this threshold excludes the Gaussian “white noise” component from the signal (Supplementary Figure 2). Events recorded at the same time frame (temporal resolution: 0.04 ms) by different microelectrodes were ascribed to the same neuron and thus counted as one event, although avalanches results were not significantly affected by this correction.

For both post stimulus and basal (resting state) activity, an average inter event interval (〈IEI〉) was calculated and used for temporal binning to estimate avalanches. Since the 〈IEI〉 is calculated in the periods post-stimuli separately from the periods of resting state, our procedure is analogous to *adaptive binning* (Yu et al., 2017). Avalanches were defined as sequences of 〈IEI〉 time bins presenting activity in the form of events, with the end of the avalanche identified by the first empty bin. The number of events in each avalanche accounted for its size, while the duration was the number of temporal bins comprising the avalanche.

### 2.3. Power Law Fitting and Statistical Testing

The avalanches sizes and durations distributions are fitted using the maximum likelihood method. The fitting function for both avalanche sizes and duration is a discrete power-law:

(1) $$\begin{array}{l} p\left(y;\alpha \right)=\frac{y^{-\alpha }}{\sum_{x=x_{min}}^{x=x_{max}}x^{-\alpha }}. \end{array}$$

The parameter *x*_*max*_ is set to the maximum observed size or duration. Then the tails of the distributions are fitted by selecting as parameter *x*_*min*_ the one that minimizes the Kolmogorov-Smirnov distance (KS), following the method proposed by Clauset et al. (2009):

(2) $$\begin{array}{l} KS=\underset{y\geq x_{min}}{\max}|S\left(y\right)-\hat{P}\left(y\right)| \end{array}$$

where *S*(*y*) is the cumulative distribution function (CDF) of the data and $\hat{P}\left(y\right)$ is the CDF of the theoretical distribution fitted with the parameter that best fits the data for *y* ≥ *x*_*min*_.

After finding the best-fit power law, to assess goodness-of-fit we compared the experimental data against 1,000 surrogate datasets drawn from the best-fit power law distribution with the same number of samples as the experimental dataset. The deviation between the surrogate datasets and a perfect power law was quantified with the KS statistic. The *p*-value of the power-law fit was defined as the fraction of these surrogate KS statistics which were greater than the KS statistic for the experimental data. Note that the data were considered power law distributed if the null hypothesis could not be rejected, namely if the the *p*-value turned out to be greater than the significance level, which was set to a conservative value of 0.1.

However, when estimating the parameters and evaluating the *p*-value, we take into consideration another aspect that has been recently pointed out in Gerlach and Altmann (2019). A point often ignored is that maximum likelihood methods rely on two assumptions:

1. the observations *y* are distributed as *p*(*y*; α), where α is the power law exponent;
2. the empirical observations *y*_*i*_, *i* = 1, …, *N*, are independent.

While the first assumption corresponds to our choice of a statistical law, statistical tests rely on the second one, which for instance is implicitly assumed when the log-likelihood is computed as $\overset{i=N}{\underset{i=1}{\sum }}\log p\left(y_{i}\right)$. However, complex systems are often characterized by strong temporal and spatial inter-dependencies, thus often violating the independence assumption. This may lead to false rejections of the statistical laws and to over-optimistic uncertainties of the estimated parameters. The authors of Gerlach and Altmann (2019) propose a method to distinguish between these assumptions, and we exploit it here to estimate the parameters and evaluate the goodness-of-fit. Briefly, we take the timeseries of sizes or durations of consecutive avalanches, and we estimate the time τ^*^ after which two observations (e.g., the avalanche sizes) are independent from each other. In practice, τ^*^ is obtained by computing the time at which the autocorrelation of the timeseries reaches an interval around zero (1-percentile of the random realization). Then, the original sequence of length *N* is randomized, N^*^ = *N*/τ^*^ observations are selected and the standard statistical analysis is applied to the new sample. This guarantees that the new sample of dimension N^*^ < *N* comprises only uncorrelated avalanches.

Indeed, in our timeseries of sizes and durations we find non negligible values of τ^*^ (see Supplementary Figure 4), and we verify that the acceptance rate of the statistical law increases for uncorrelated avalanches. Because of the variability of the different realizations of the subsampling procedure, the exponents and the *p*-values shown in Tables 1, 2 are obtained averaging over 20 repetitions of this subsampling.

**Table 1:** Exponents before and after subsampling in LFP data, with the corresponding *p*-values.

		**τ**	***p*-values**	**τ (sub.)**	***p*-values (sub.)**	**τ_*t*_**	***p*-values**	**τ_*t*_ (sub.)**	***p*-values (sub.)**
Rat 1	Post stim.	1.85	0.04	1.87	0.48	2.44	0.16	2.45	0.50
	Resting state	1.82	<0.001	1.80	0.33	2.38	0.40	2.36	0.63
Rat 2	Post stim.	1.59	0.1	1.59	0.50	1.87	0.004	1.88	0.14
	Resting state	1.60	0.02	1.60	0.38	1.84	<0.001	1.84	0.24
Rat 3	Post stim.	1.60	0.13	1.60	0.41	2.19	0.05	2.18	0.36
	Resting state	1.57	0.07	1.58	0.60	1.87	<0.001	1.87	0.03
Rat 4	Post stim.	1.98	0.27	1.97	0.56	2.78	0.41	2.75	0.7
	Resting state	2.16	0.54	2.15	0.72	2.67	0.30	2.65	0.58
Rat 5	Post stim.	1.74	0.30	1.73	0.71	2.19	0.07	2.19	0.45
	Resting state	1.72	0.56	1.73	0.78	2.18	0.09	2.20	0.55

*Note that the p-values obtained after subsampling are always greater than the significance level 0.1 both for τ and τ_t_, except for the τ_t_ p-value of rat 3*.

**Table 2:** Exponents before ad after subsampling in MUAs data, with the corresponding *p*-values.

		**τ**	***p*-values**	**τ (sub.)**	***p*-values (sub.)**	**τ_*t*_**	***p*-values**	**τ_*t*_ (sub.)**	***p*-values (sub.)**
Rat 1	Post stim.	1.98	0.077	1.97	0.63	2.27	0.27	2.30	0.79
	Resting state	2.23	0.2	2.23	0.66	2.52	0.34	2.52	0.67
Rat 2	Post stim.	2.00	0.73	1.95	0.74	2.25	0.41	2.27	0.74
	Resting state	2.04	0.003	2.03	0.43	2.23	<0.001	2.23	0.1
Rat 3	Post stim.	2.05	0.72	2.04	0.75	2.34	0.84	2.29	0.73
	Resting state	2.18	0.004	2.18	0.42	2.41	<0.001	2.41	0.26
Rat 4	Post stim.	1.98	0.06	2.00	0.91	2.51	0.01	2.33	0.63
	Resting state	2.50	0.12	2.50	0.19	2.67	<0.001	2.60	<0.001

*Note that the p-values obtained after subsampling are always greater than the significance level 0.1 both for τ and τ_t_, except for the τ_t_ p-value of rat 4*.

## 3. Results

### 3.1. Preliminary Considerations

The standard approach to infer criticality is to search for neuronal avalanches whose sizes and durations follow scale-free distributions in resting state (i.e., unperturbed) conditions. Indeed, at criticality, it is expected that such distributions scale as the power laws

(3) $$\begin{array}{l} P\left(S\right)\sim S^{-\tau }, \end{array}$$

(4) $$\begin{array}{l} P\left(T\right)\sim T^{-\tau_{t}}, \end{array}$$

where *S* is the number of events in an avalanche (i.e., the size), *T* is its duration (also called avalanche lifetime) and τ and τ_*t*_ are the related critical exponents. Power laws, however, can also stem from non critical systems and generative mechanisms. Thus, a more robust test of criticality is to verify whether the so-called crackling noise relation holds. This scaling relation was first developed in the context of crackling noise (Sethna et al., 2001), hence the name, but nonetheless it is expected to hold in general in all systems close to their critical point (Friedman et al., 2012), and in particular in systems with absorbing states (di Santo et al., 2017). The relation predicts that the critical exponent δ, which relates the duration of an avalanche to its mean size as

(5) $$\begin{array}{l} \langle S\rangle \left(T\right)\sim T^{\delta_{\text{fit}}} \end{array}$$

obeys the scaling relation

(6) $$\begin{array}{l} \delta_{\text{pred}}=\frac{\tau_{t}-1}{\tau -1}. \end{array}$$

We estimated δ in two independent ways, as δ_pred_ and as δ_fit_, i.e., the slope of the least square fit of the average sizes given their durations. In principle, if these two estimates are compatible, then the system is compatible with criticality. Proving such relation is however challenging. First, it is sensitive to the fitting methods of the distributions of avalanche sizes and lifetimes, as it has been recently shown (Destexhe and Touboul, 2021). Second, in the case of LFPs, the range of avalanche lifetimes typically extends over one order of magnitude only, which undermines reliability of power law fitting.

A complementary approach is to look for criticality by perturbing the system, that is by shifting activity to a subcritical or supercritical regime and then measure the distance from a critical state (Shew et al., 2009; Meisel, 2020). Thus, when measuring neural activity across a cortical barrel column of the rat brain, we also provided sensory stimuli consisting of impulsive deflections of the corresponding whisker and therefore representing strong, well defined, and accurately reproducible perturbations.

Neural avalanches sizes and durations were fitted with a discrete power law through the maximum likelihood method (see section 2.3). As mentioned in the Materials and Methods section, we found non negligible values of the correlation time τ^*^ both for spikes/MUAs and LFPs, suggesting that events were not independent. Therefore, to test if avalanches were power law distributed, we corrected for dependencies between avalanches by subsampling the data of sizes and durations as described in section 2.3. Further results are reported in Supplementary Tables 1–8.

### 3.2. Avalanches in LFP Data

**Figure 3:**
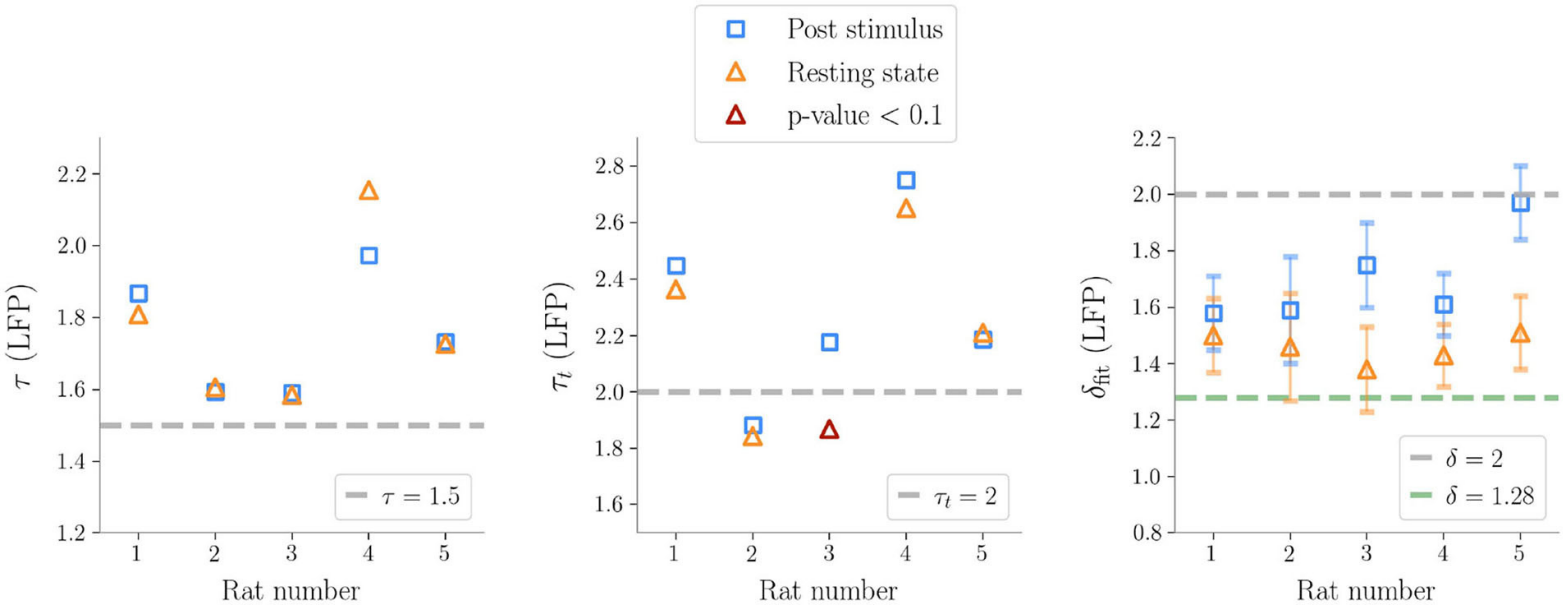
Exponents obtained after subsampling in Local Field Potential data (τ is the exponent for the sizes, τ_*t*_ for the durations, δ_fit_ is the fitted exponent from $\text{S}\left(\text{T}\right)\sim T^{\delta_{\text{fit}}}$). The exponents predicted by the critical branching process are shown in the plot as gray dashed lines. The line δ = 1.28 is also plotted, which is reported in Fontenele et al. (2019) as a universal exponent found in many different experiments. It is noteworthy that, as seen in the plot on the right, the exponents δ_fit_ are always far from the hallmark of the branching process, except for one case after stimulation, where the presence of large bumps in the size distribution makes the slope of the line in the (〈*S*(*T*)〉, *T*) plane steeper.

We first analyzed avalanches in LFP recordings and found similar results across five animals (Table 1). First of all, we focused on spontaneous (i.e., resting, non stimulated) activity. We estimated τ^*^ and subsampled the sizes and the lifetimes accordingly to ensure that individual observations were uncorrelated (thus not violating the assumption of the maximum likelihood method). Following this correction, avalanches resulted power law distributed across the five rats, except for one case for the avalanches durations (see Figure 3 and Table 1).

**Figure 4:**
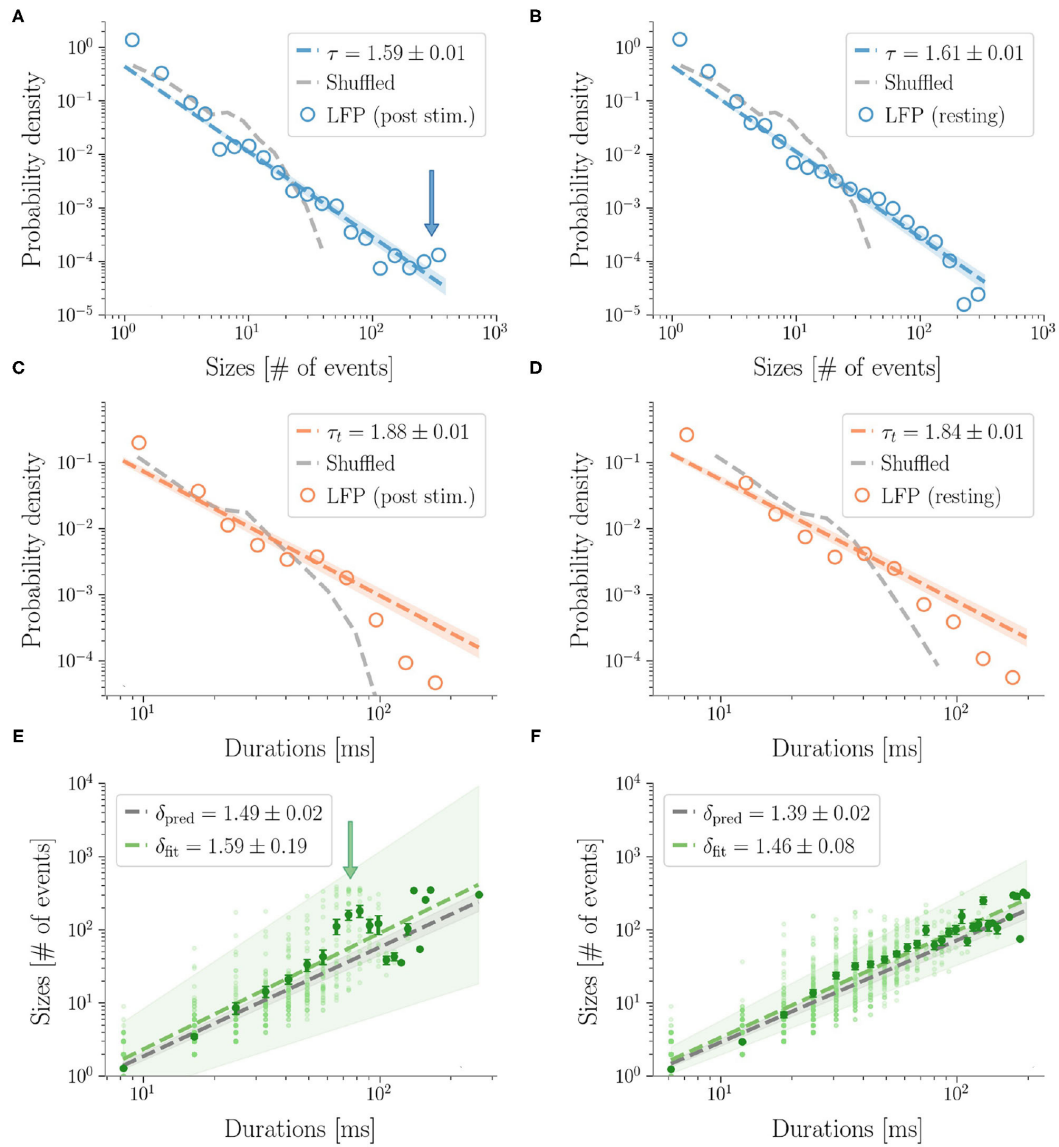
LFP avalanches. Representative probability densities of avalanches sizes in one rat after the sensory stimulus **(A)**, or in resting state conditions **(B)**. A bump deviating from the power law can be recognized in the post-stimulus distribution in correspondence of large avalanche sizes, pointed to by an arrow. Distributions of the avalanches' durations do not display clear alterations instead **(C,D)**. Durations are expressed in *ms* by multiplying the number of bins by 〈IEI〉. In the randomized dataset the exponential distribution provides a better fit in all cases (dashed gray lines). The shuffling procedure consists in randomizing the occurrence times of the events of each channel, so that the events rate of each channel is preserved. **(E,F)** The crackling noise relation is verified in both cases within the experimental errors (i.e., δ_pred_ is compatible with δ_fit_). Once more, notice in **(E)** the presence of a bump (indicated by an arrow) in the post-stimulus regime.

Then, we analyzed stimulus-evoked responses over 2 s after the stimulus and confirmed power law scaling (accepted by statistical tests). However, at a more careful look, the size distribution was altered by the presence of a *bump* and similar to the heap observed in previous work (di Santo et al., 2018) (Figure 4). Clearly, the bump derived from an excess of large size avalanches (i.e., involving a large number of microelectrodes). As suggested by Figure 2, these avalanches were not only large, but also composed by highly synchronous events across cortical layers. In fact the bump vanished in the duration distribution, confirming that the size increase was not accompanied by a corresponding increase of lifetime and that events remained concentrated within a few time bins.

In general, we found that the crackling noise relation was verified both at resting and during the post-stimulus (Figure 4 and see Supplementary Figure 5 for the avalanches results on all the rats). However, after the stimulus, a localized deviation from the expected trend was observed in correspondence of the bump found in the size distribution, and therefore also caused by large and synchronized waves of activity unleashed by whisker deflection. As anticipated previously, avalanches durations, and consequently also 〈*S*〉(*T*), extended over about two orders of magnitude. In fact, since LFPs are average signals that integrate over space and time single neuron events, it is expected that the maximum size of the avalanches extracted from LFPs is of the order of the array size (see Supplementary Figure 3 for an analysis of finite size effects in LFPs avalanches) (Beggs and Plenz, 2003). Specifically, a cutoff in *P*(*S*) around a value *N*_*C*_ ≈ *N*_*E*_, with *N*_*E*_ the number of the recording electrodes, is commonly observed experimentally, meaning that during an avalanche each electrode is typically activated just once. This cutoff in *P*(*S*) implies that 〈*S*〉(*T*) < *N*_*C*_, and, from 〈*S*〉(*T*) ~ *T*^δ^, it also implies $T<N_{C}^{\frac{1}{\delta }}$ (Neto et al., 2020). Thus, if δ > 1, the cut-off in *P*(*S*) causes a much earlier cut-off in both *P*(*T*) and 〈*S*〉(*T*).

### 3.3. Avalanches in Spikes and MUAs Data

Spikes and MUAs avalanches (largely dominated by MUAs as reported in the Methods) were analyzed for four rats. Albeit similar to LFPs, the results unveiled important differences. First of all, MUAs were much less affected by the finite size of the recording array. In fact, despite the low number of microelectrodes (twenty-seven sites spanning across the cortex in the vertical direction), avalanches could be observed with size greater than 10^2^ events. The reason is that, in MUAs, the same avalanche can reach an electrode repeatedly and in quick succession, contrary to LFPs where single neuron contributions are integrated over space and time within the brain tissue and coalesce to generate the recorded signal (Neto et al., 2020). As a consequence, MUAs provided a much better estimate of the avalanche duration distributions.

**Figure 5:**
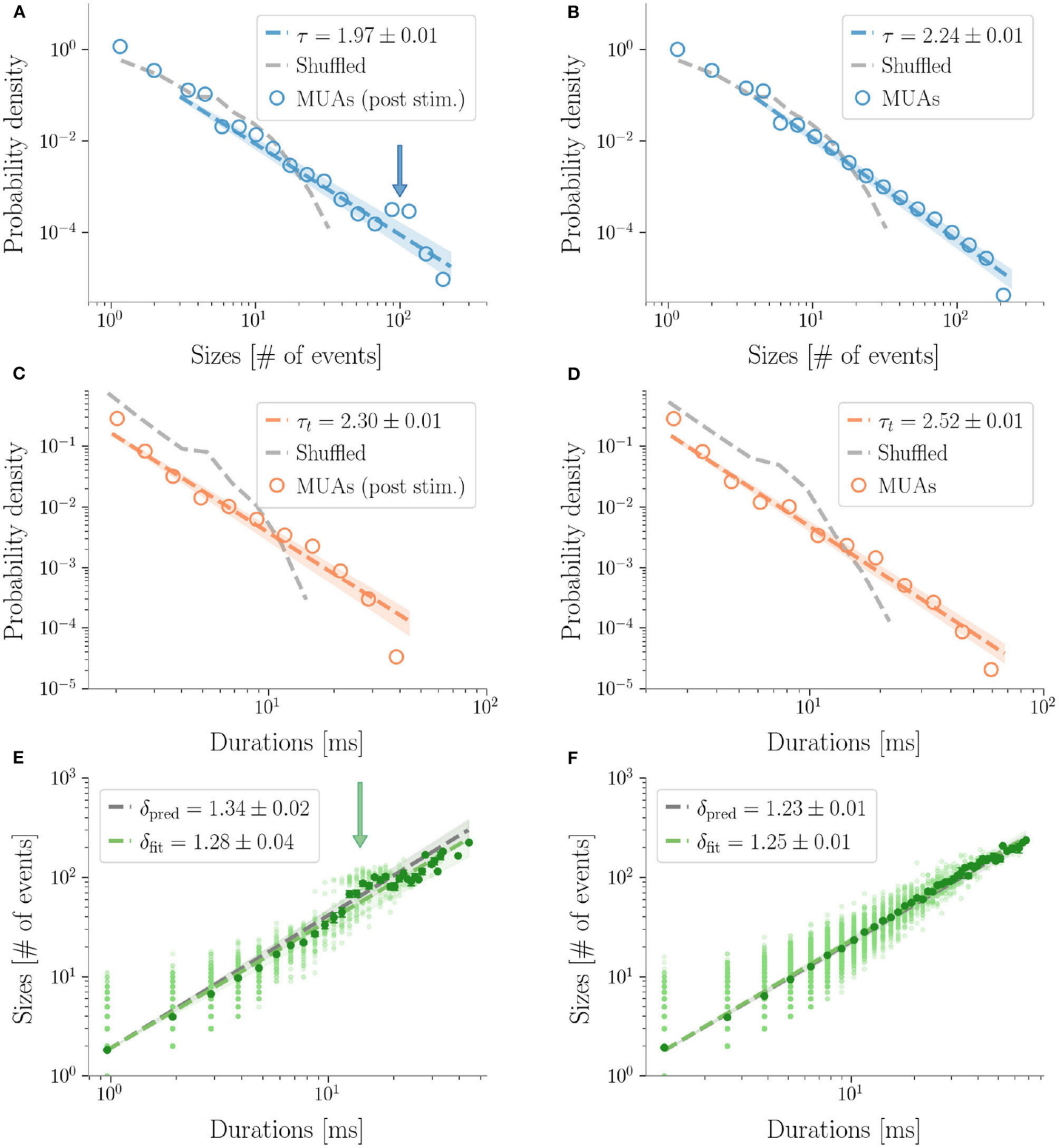
Avalanches in MUAs data. Avalanches sizes probability density in one rat after a stimulus **(A)**, and during resting state **(B)**. Notice the bump in the post-stimulus distribution. **(C,D)** The same, for avalanche durations. Durations are expressed in *ms* by multiplying the number of bins times 〈IEI〉. In the randomized datasets the exponential distribution provides a better fit in all cases. The shuffling procedure consists in randomizing the occurrence times of the events of each channel, so that the events rate of each channel is preserved. **(E,F)** The crackling noise relation is verified in both cases within the experimental errors. Once more, notice in **(E)** the presence of a bump in the post-stimulus regime.

**Figure 6:**
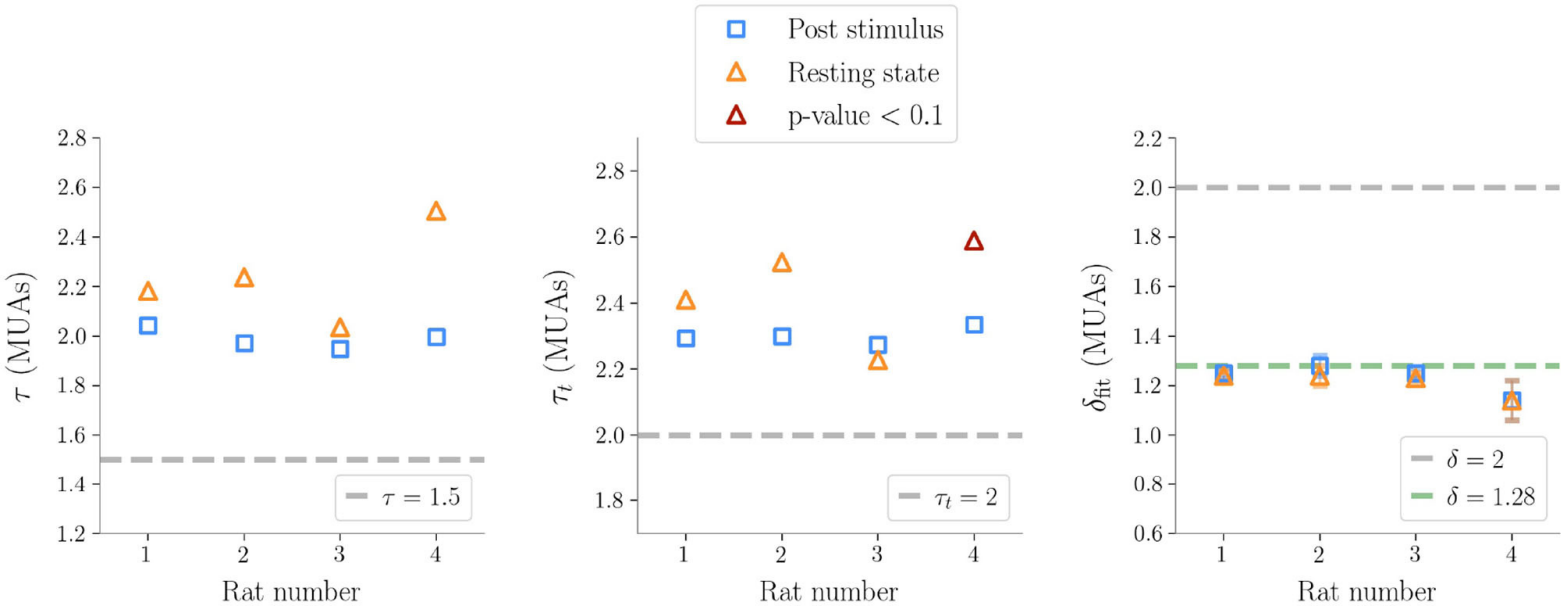
Exponents obtained after subsampling in multi-unit activities data (τ exponent for the sizes, τ_*t*_ for the durations, δ_fit_ the fitted exponents of the crackling-noise relation). The exponents predicted by the critical branching process are also plotted. The line δ_fit_ = 1.28 is also plotted, which is found in Fontenele et al. (2019), and which turns out to be remarkably close to our results.

Similarly to LFPs, we found a power law distribution of avalanches both in terms of size and duration, except for one rat deviating from this general trend with respect to duration alone (**Figure 6** and Table 2). Moreover, we confirmed the emergence of the bump of activity in the post-stimulus size distribution caused by the abundant number of large-sized avalanches (Figure 5). The exponents τ and τ_*t*_ were greater than the ones found in LFPs (Table 2), but also greater than the ones predicted for a critical branching process (Figure 6). Interestingly, the exponent δ was consistently close to the value δ ≈ 1.28 which was found in Fontenele et al. (2019) to be universal, i.e., to hold across different experimental conditions, from cultured slices to freely moving or anesthetized mammals.

Finally, as avalanche size and duration were less affected by finite size effects, MUAs allowed us to reliably test the crackling noise relation, which was verified in all dataset (Figure 5, see also Supplementary Figure 6). In particular, as MUAs data are believed to be more robust in this respect (Friedman et al., 2012), we concluded that post-stimulus avalanches statistics is also compatible with the results recently reported by Fontenele and collaborators (Fontenele et al., 2019). Nevertheless, also in this case, the waves of large and synchronized activity triggered by whisker deflection generated a local bump deviating from the crackling noise relation.

## 4. Discussion and Conclusions

In this work we investigated criticality in the rat barrel cortex, which offers advantages over other sensory systems. First, there is a clear and well characterized somatotopic representation of the whiskers in this primary sensory cortex, where single whiskers are mapped to single cortical columns with a one-to-one correspondence. This differentiates the barrel cortex from, e.g., the primary visual cortex, where functional columns are not as clearly segregated by septa in a cylindrical structure perpendicular to the cortex surface as in the barrel field, which facilitates the recording of single column activity by a neural probe (Horton and Adams, 2005). Second, whisker receptors, which are transducers placed around the follicle, directly activate primary sensory neurons, that therefore encode whisker deflections without interposed processing. In other circumstances, such as in the visual system, the transduced stimulus is subject to extensive processing already at the periphery (e.g., by the retina network) which makes it difficult to disentangle the dynamics and the contributions of cortical and pre-cortical networks in response to sensory inputs (Diamond et al., 2008; Feldmeyer et al., 2013). Third, single-whisker deflection can be controlled with high accuracy through closed-loop piezoelectric systems enabling a tight experimental control over repeated sensory stimuli.

As for other sensory cortical areas, it can be hypothesized that the barrel cortex takes advantage of criticality to efficiently map tactile stimuli. This holds also for the single barrel column, which faces the severe challenge to represent the parameters related to deflection of its corresponding whisker (e.g., amplitude, direction and velocity of displacement), as transduced by the follicle receptors, in an efficient, noise-tolerant manner and in real-time. Along a similar line of reasoning, previous work has addressed the barrel cortex (Gautam et al., 2015) using an air puff on the snout for stimulation and with avalanches analysis that was tailored to UP/DOWN states dominating activity under urethane anesthesia. According to a simplified general model of the processing in the barrel column, it is believed that layer IV acts as main input stage of sensory information propagating from the thalamus, whereas layer V is the main output. However, the few thousands neurons composing a single barrel form complex microcircuits of excitatory and inhibitory connections across layers. These microcircuits generate a rich dynamics that has been shown, both *in-vitro* and *in-vivo*, to include avalanches or synchronization states, such as UP and DOWN states and oscillations, and whose significance in terms of tactile information mapping and processing is far from being understood (Feldmeyer, 2012; Petersen, 2019).

We run our experiments under tiletamine anesthesia by precisely controlling single whisker deflection by a closed-loop controlled piezoelectric actuator. With tiletamine, that contrary to urethane is also used in clinics, spontaneous activity remains rich, with a mix of oscillations and UP and DOWN states, and with the activity evoked by whisker stimulation that shows clear similarities to the one in the awake animal (Rojas et al., 2006). In the context of our study, another advantage of the anesthetized animal with respect to the awake condition was that, by reducing cortico-cortical communication, the anesthetic was insulating the somatosensory barrel region from external “contaminating” waves of activity propagating from other brain areas (Aronoff et al., 2010; Voss et al., 2019; Sorrenti et al., 2021).

We measured neural activity across the six cortical layers of a single barrel both in the domain of spikes/MUAs (although MUAs were largely overwhelming) and LFPs, thus extending the avalanches analysis over a wide frequency range and covering both single neuron and population dynamics. Recently, some first attempts were made to characterize criticality in neural systems in a broader sense (Agrawal et al., 2019; Meshulam et al., 2019; Nicoletti et al., 2020) but, despite some well-known limitations (Beggs and Timme, 2012), as of now scale-free avalanches are still the most employed approach to test the critical brain hypothesis. For LFPs, we used a high-density 2D array of microelectrodes developed at the purpose to monitor at high spatial resolution the electrical potential within a planar section of the barrel column (Thewes et al., 2016) whereas for spikes and MUAs we used a 32 channels linear array probe.

On the one hand, and contrary to previous works based on imaging methods (Scott et al., 2014; Bellay et al., 2015), we found that in the anesthetized animal avalanche sizes and lifetimes followed power laws during basal activity, in agreement with findings on LFPs and spikes (Hahn et al., 2010; Ribeiro et al., 2010). Notably, we observe inter-rats variability in the power-law exponents (Figures 3, 6), but this phenomenon is not unexpected as it was observed in previous experiments (Shew et al., 2015) and a theoretical explanation has also been recently proposed in terms of quasi-criticality (Fosque et al., 2021). In particular, in Fontenele et al. (2019) a statistical meta-analysis of a large number of experiments suggests that, even if the avalanche exponents vary across individuals and across experimental settings, they all lie along a scaling line consistent with the crackling noise relation and an exponent δ ≈ 1.28. We do find that the exponent δ consistently converge to the same value (Figure 6). Intriguingly, and in contrast to the classical view of avalanches seen as generated by a quiescent-to-active phase transition—even though a different scenario involving a disorder-induced transition has been recently proposed as well (Ponce-Alvarez et al., 2018)—the analysis of Fontenele et al. (2019) suggests that the critical transition occurs at the edge of synchronization (di Santo et al., 2018) and this non-trivial exponent might emerge in different contexts (Dalla Porta and Copelli, 2019; Neto et al., 2020; Carvalho et al., 2021). In this context, we also note that if we assume as a generative mechanism a model with absorbing states, such as the branching process, it would imply no correlation between avalanches (Dalla Porta and Copelli, 2019). However, in our data, we found non-negligible values of τ^*^ hinting at the existence of temporal correlations between subsequent avalanches. These latter results are in agreement with previous works in which long range temporal correlations emerged through a detrended fluctuations analysis (DFA) of the signals (Linkenkaer-Hansen et al., 2001; Ribeiro et al., 2010; Hardstone et al., 2012). Although the nature of such correlations remains unknown, we speculate that they may derive from a variety of neuronal and circuital mechanisms including, e.g., a neuromodulation of the barrel column, acting at time scales that are large compared to avalanches lifetimes.

After the stimulus, power laws displayed localized bump-like alterations. The associated Kuramoto order parameters (see Supplementary Table 9) point at the emergence of a high, but brief, synchronization across layers. A control test of the avalanches' distributions without the first 200 ms after the stimulus shows indeed that the bumps in the sizes' distributions disappear both in LFPs and MUAs (see Supplementary Figure 7). Importantly, bumps are not just a spurious effect of the bin chosen, as we adopted a similar procedure to the one proposed by Yu et al. (2017) by evaluating 〈IEI〉 in the periods after stimuli separately from the periods of resting state. However, at the same time, one should note that bumps are also influenced by the finite size of the system (de Arcangelis, 2012). Indeed, being bounded by the system size, an excess of large avalanches can create a peak at a characteristic scale in the distributions. This is precisely the case of LFP avalanches, that are known to be constrained by the size of the array. In the case of MUAs, instead, finite size constraints are more relaxed, bumps are genuine although less evident in some MUAs distributions (see Supplementary Figure 6). Hence, in summary, the presence of bumps marks the occurrence of avalanches with large sizes that appear with a higher probability than what predicted by the power law scaling and, in the case of LFPs, these sizes are of the order of the array size.

This seemingly simple observation has quite profound implications. These avalanches, characterized by a highly synchronous activity across microelectrodes (see Figure 2), are related to a strong response to the stimulus that is thought to emerge prominently in layer IV first, and then to quickly spread among the other layers eliciting a global activity in the barrel. This suggests that synchronization waves together with oscillations play a fundamental role in shaping the neural activity during the mapping of the tactile stimulus. Indeed, the LFPs evoked response is also characterized by oscillations at a frequency close to 6 Hz (Supplementary Figure 14), whose power is particularly high in the central layers. These oscillations are not present in the spontaneous activity phase. Let us note that, most likely, these small-amplitude oscillations in the LFP mainly reflect subthreshold synaptic potentials as the 6 Hz component is not visible in the spikes domain (see Supplementary Figure 14). Moreover, being below the signal detection threshold for LFPs, these oscillations do not overall contribute to LFP avalanches distributions. In recent years, some modeling efforts taking into account synchronizations and oscillations phenomena were made (Poil et al., 2012; di Santo et al., 2018; Fontenele et al., 2019) but a comprehensive model of this kind remains elusive and further experimental studies will be needed to address this crucial point.

In perspective, in addition to further investigating the relation between resting state and stimuli with respect to brain criticality, an interesting route could be the investigation of other markers of criticality beyond neuronal avalanches, such as scale-free correlations (Mariani et al., 2021) or other patterns of dynamic functional connectivity, e.g., obtained by computing the instantaneous phase difference between signals at different locations (Cabral et al., 2017). On the other hand, it is likely that sound models of the possible self-organizing mechanisms of the cortex will have to take into account its oscillatory behavior, whose signature in the present work emerges through both the bumps in the avalanche size distribution and the presence of a characteristic peak in the power spectrum in the periods after stimuli. In perspective, avalanches and oscillations may represent two intertwined and functionally relevant faces of the cortical brain dynamics, that will have to be considered together in future theoretical and experimental investigations on the brain's sensory coding.

## Data Availability Statement

The raw data supporting the conclusions of this article will be made available by the authors, without undue reservation.

## Ethics Statement

The animal study was reviewed and approved by Italian Ministry of Health.

## Author Contributions

SV and SS designed the study. SV, SS, and MB supervised the research. MM and AL conducted the experiments. RO designed the whisker control system. BM analyzed the data. BM, GN, MB, MM, SS, and SV interpreted the results and wrote the manuscript. BM and GN prepared the figures. All authors contributed to the article and approved the submitted version.

## Conflict of Interest

The authors declare that the research was conducted in the absence of any commercial or financial relationships that could be construed as a potential conflict of interest.

## Publisher's Note

All claims expressed in this article are solely those of the authors and do not necessarily represent those of their affiliated organizations, or those of the publisher, the editors and the reviewers. Any product that may be evaluated in this article, or claim that may be made by its manufacturer, is not guaranteed or endorsed by the publisher.
