# Supplementary material for: Neuronal Avalanches Across the Rat Somatosensory Barrel Cortex and the Effect of Single Whisker Stimulation
Source: Front Syst Neurosci. 2021 Aug 30;15:709677. doi: 10.3389/fnsys.2021.709677 (PMC8435673; doi:10.3389/fnsys.2021.709677)
Supplement: Supplementary file 1 [file Data_Sheet_1.pdf]

Supplementary Material

1 SUPPLEMENTARY FIGURES

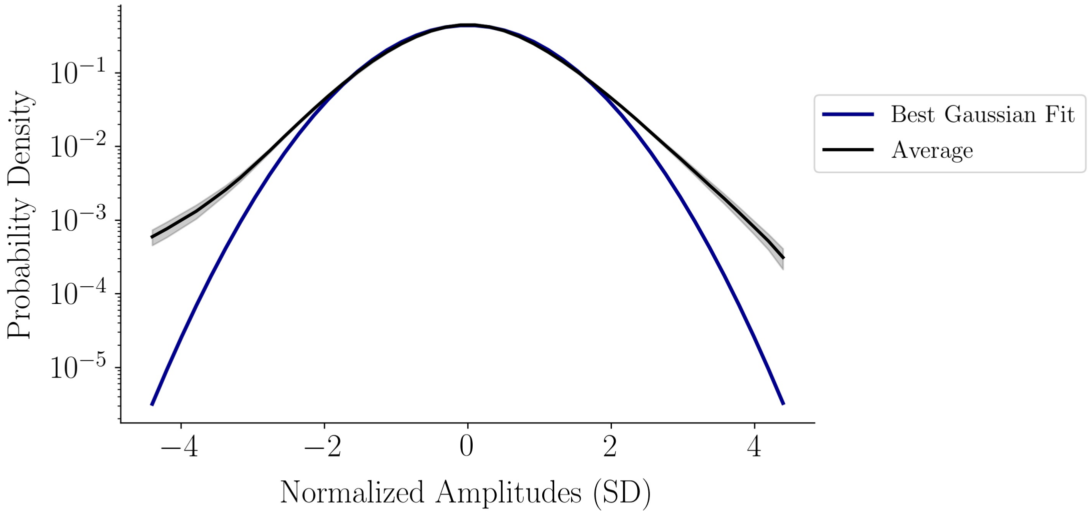

Figure S1: **LFPs events threshold definition**. The red curve depicts the grand average of the signals amplitude distributions over all channels and trials in LFPs of one rat. Note that the signal from each channel is z-normalized by subtracting its mean and dividing by the SD. The dashed line depicts the best fit of a Gaussian distribution to the data for the range between + 4.5 SD and - 4.5 SD. The Gaussian fit starts deviating from the average signal at around $\pm$ 2 SD. Hence, in order to avoid false positives, we set the event threshold at $\pm$ 3 SD. A logarithmic scale is used for the y-axis.

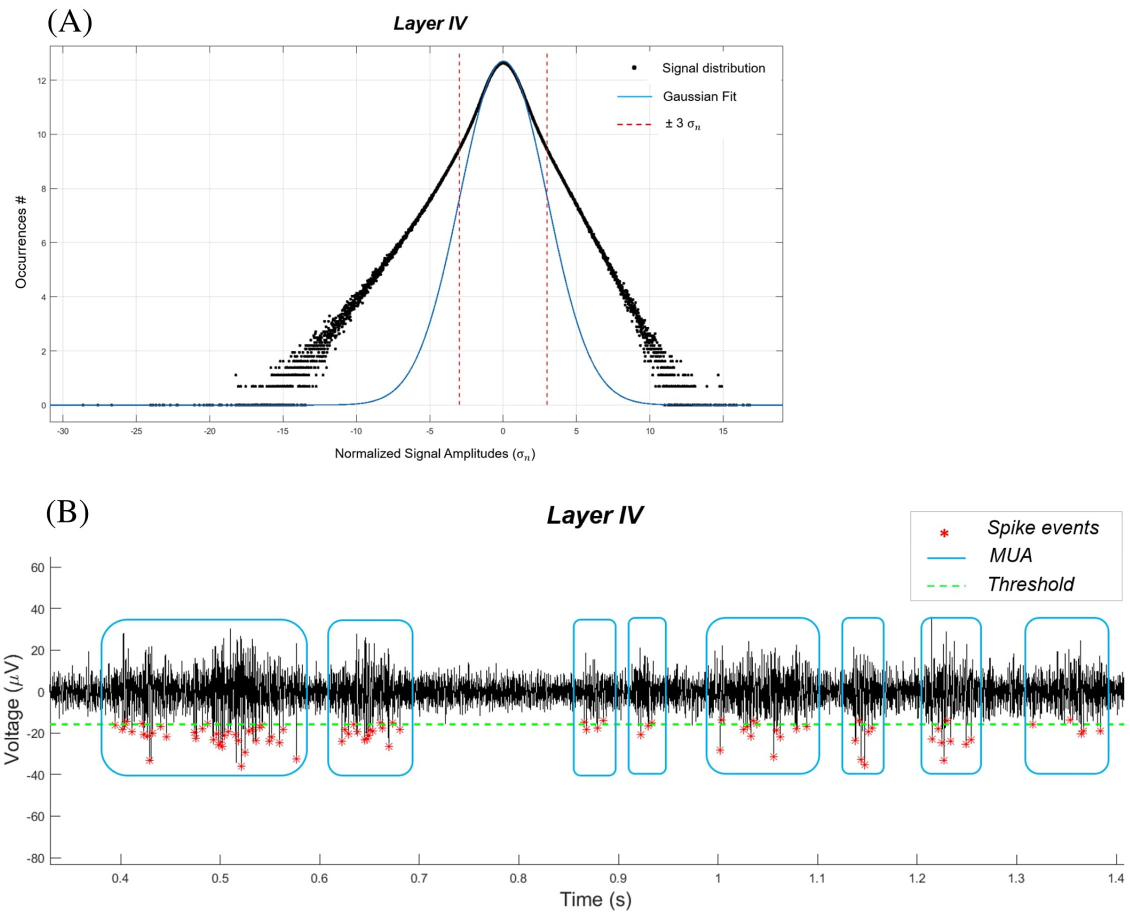

Figure S2: **MUAs activity and threshold definition.** In figure A we show the distribution of MUAs signals amplitude of a channel in layer IV. It is possible to see that the threshold chosen to detect events (3 standard deviations of the noise [1]) falls beyond the region of the distribution that is well fitted by a Gaussian. In (B) the corresponding time series is reported, together with the identified spike events.

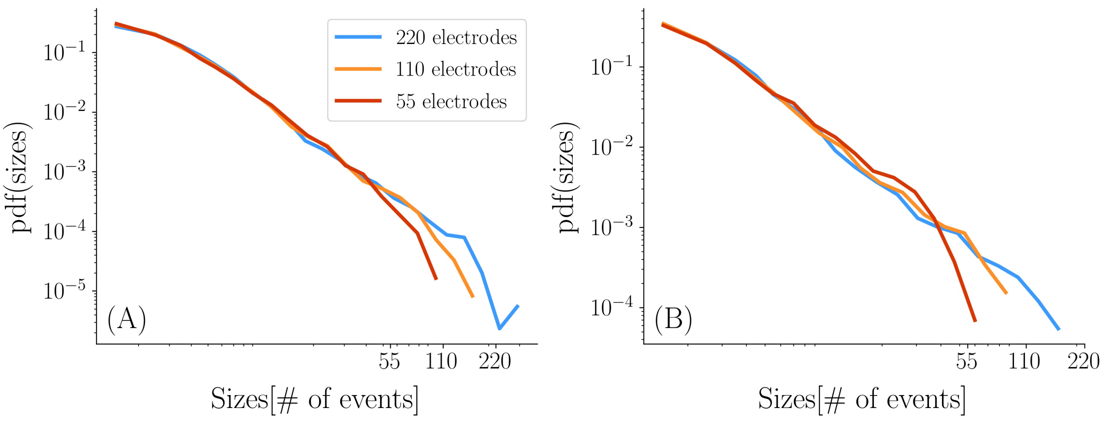

Figure S3: **Power law finite size effects analysis for LFP avalanche sizes at resting state**. We verify that the cutoff in LFP avalanches distribution is dependent on the size of the array. For this purpose, we repeat the avalanche analysis considering only halves and quarters of the array. For example, when considering quarters, only single columns of the array 55×4 are considered in the analysis: the array is split along the direction of the barrel column, in order not to create halves/quartets with different behaviors due to the inclusion of different layers. The results from the four columns are averaged to produce the analysis for a quarter of the array. The same procedure is applied to the two halves of the array. We verify that the maximum size of the avalanches (called here N_C) is dependent on the number of electrodes (N_E) of the array. It results that $N_C \geq N_E$ when considering both positive and negative excursions of the signals as events (Fig. S3 A), as in the main text [2], and it results that $N_C \approx N_E$ when only negative excursions are considered as events (Fig. S3 B). As noted in the main text, this cutoff in the avalanche sizes induces a much earlier cutoff in avalanche durations. Also, the exponents do not change when reducing the number of electrodes.

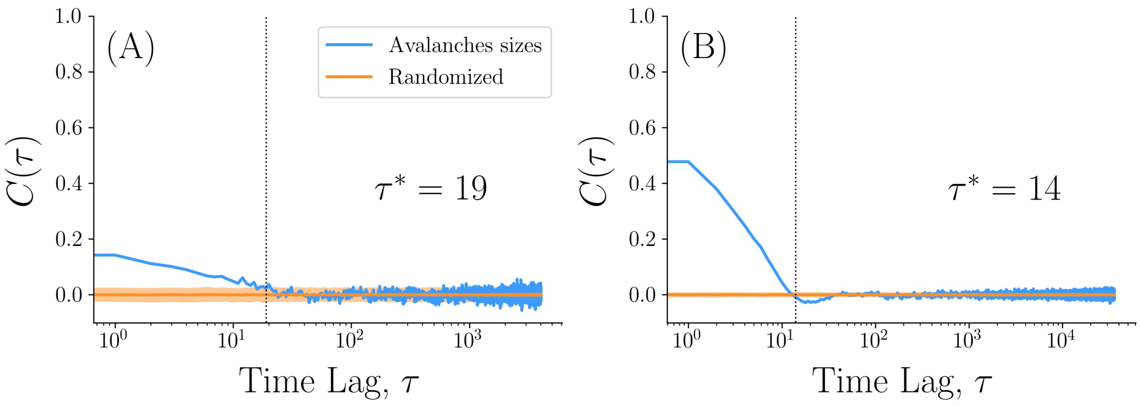

Figure S4: **Are avalanches correlated?** As these plots show, subsequent avalanches are correlated. The autocorrelation function of the logarithm of avalanche sizes during resting state is computed following [3]. (A) Avalanches in LFPs data display a characteristic autocorrelation time of $\tau^* = 19$, i.e. on average τ^* consecutive avalanches are correlated. (B) In MUAs data, $\tau^* = 14$.

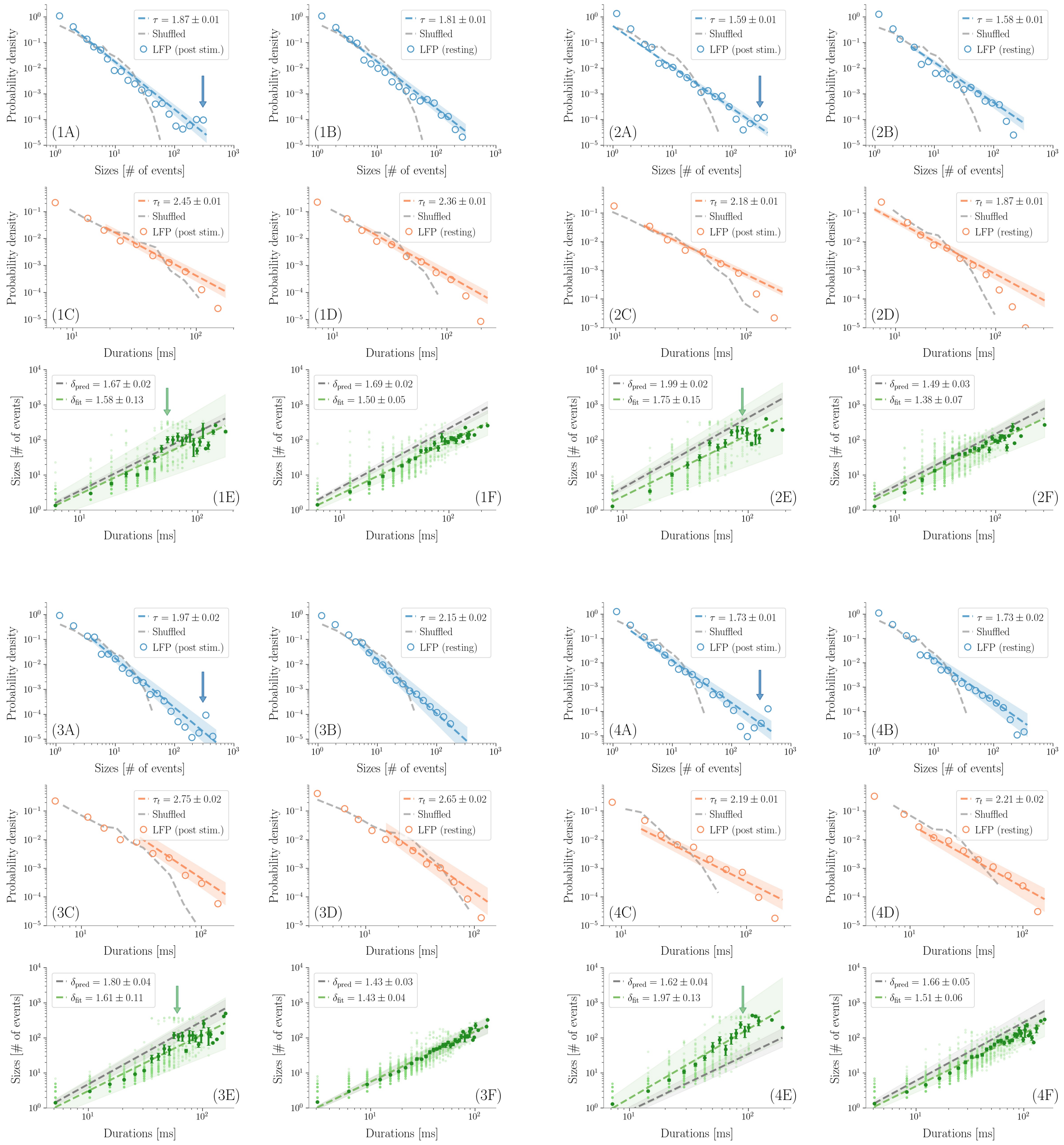

Figure S5: **Distribution of avalanche sizes (in blue), durations (in red) and crackling noise relation (in green) in LFPs data** obtained as described in the main text, both at resting and post stimulus for four different rats. Detailed avalanche statistics results are presented for all rats in the main text.

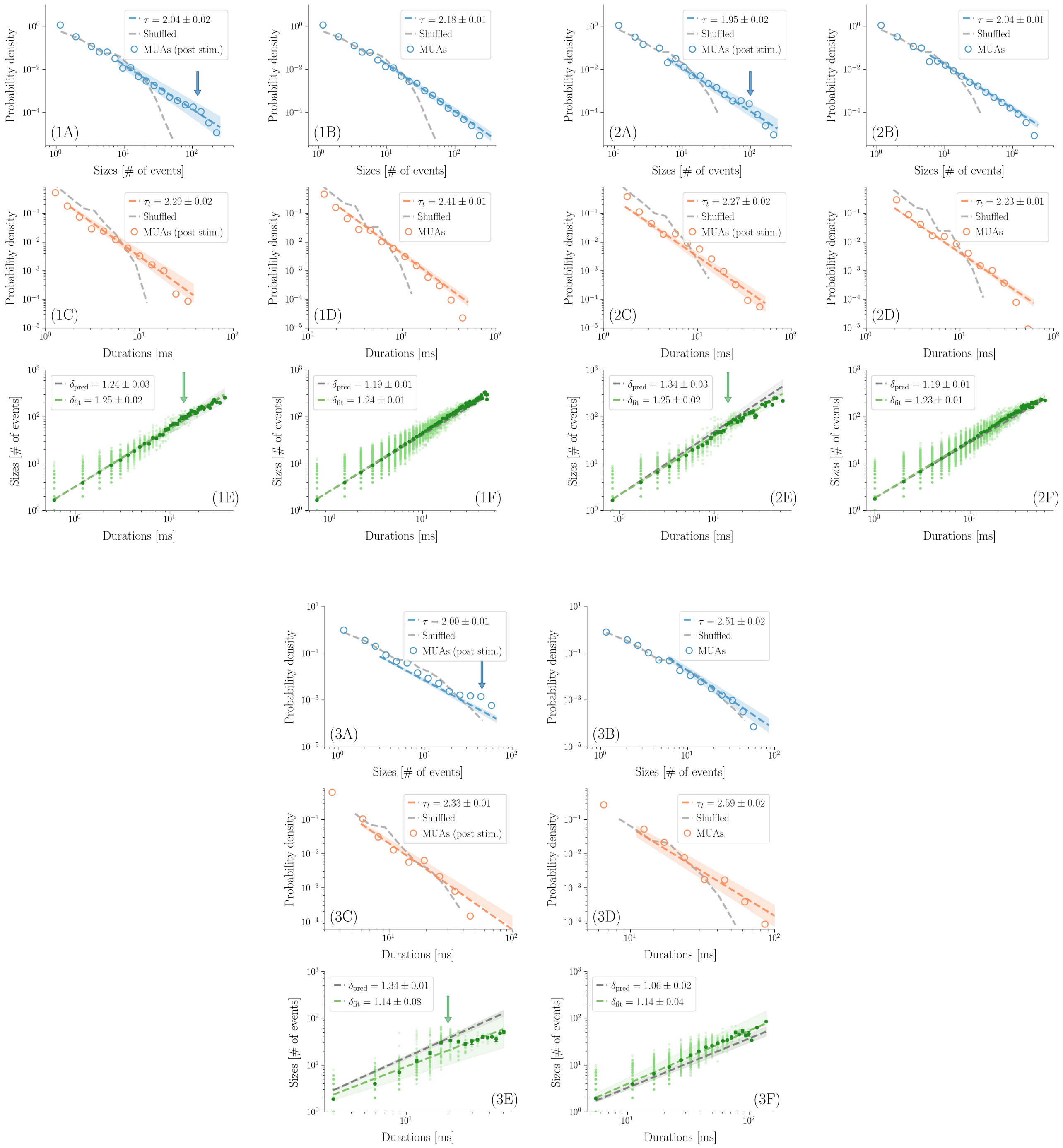

Figure S6: **Distribution of avalanche sizes (in blue), durations (in red) and crackling noise relation (in green) in MUAs data** obtained as described in the main text, both at resting and post stimulus for three different rats. Detailed avalanche statistics results are presented for all rats in the main text.

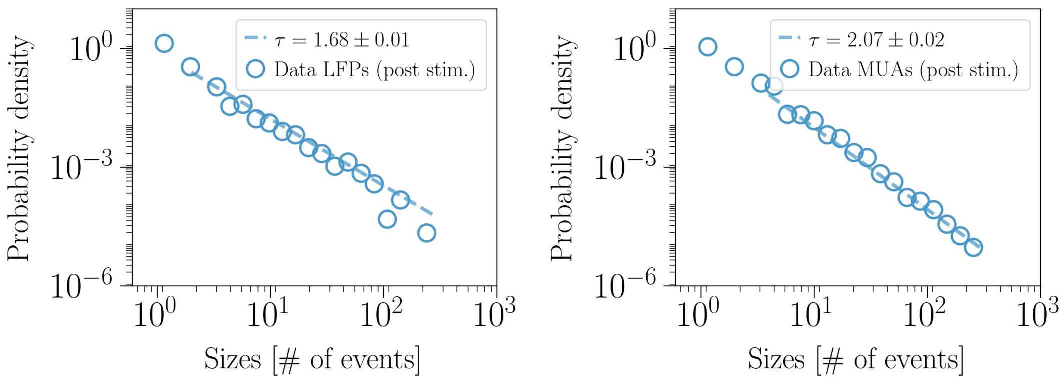

Figure S7: **Avalanches post stimulus disregarding a short transient.** In our experiments the stimulation is impulsive, leading to a strong transient response that is reflected in bumps in avalanches' sizes distributions in correspondence of large avalanches. If we disregard the first 200ms after stimulation from the analysis of the post-stimuli periods of both LFPs and MUAs, bumps in the distributions disappear and the distributions do not present anymore local deviations from the power law trend. Here we present avalanche sizes' distributions corresponding to the rats in Figures 3 and 5 of the main text.

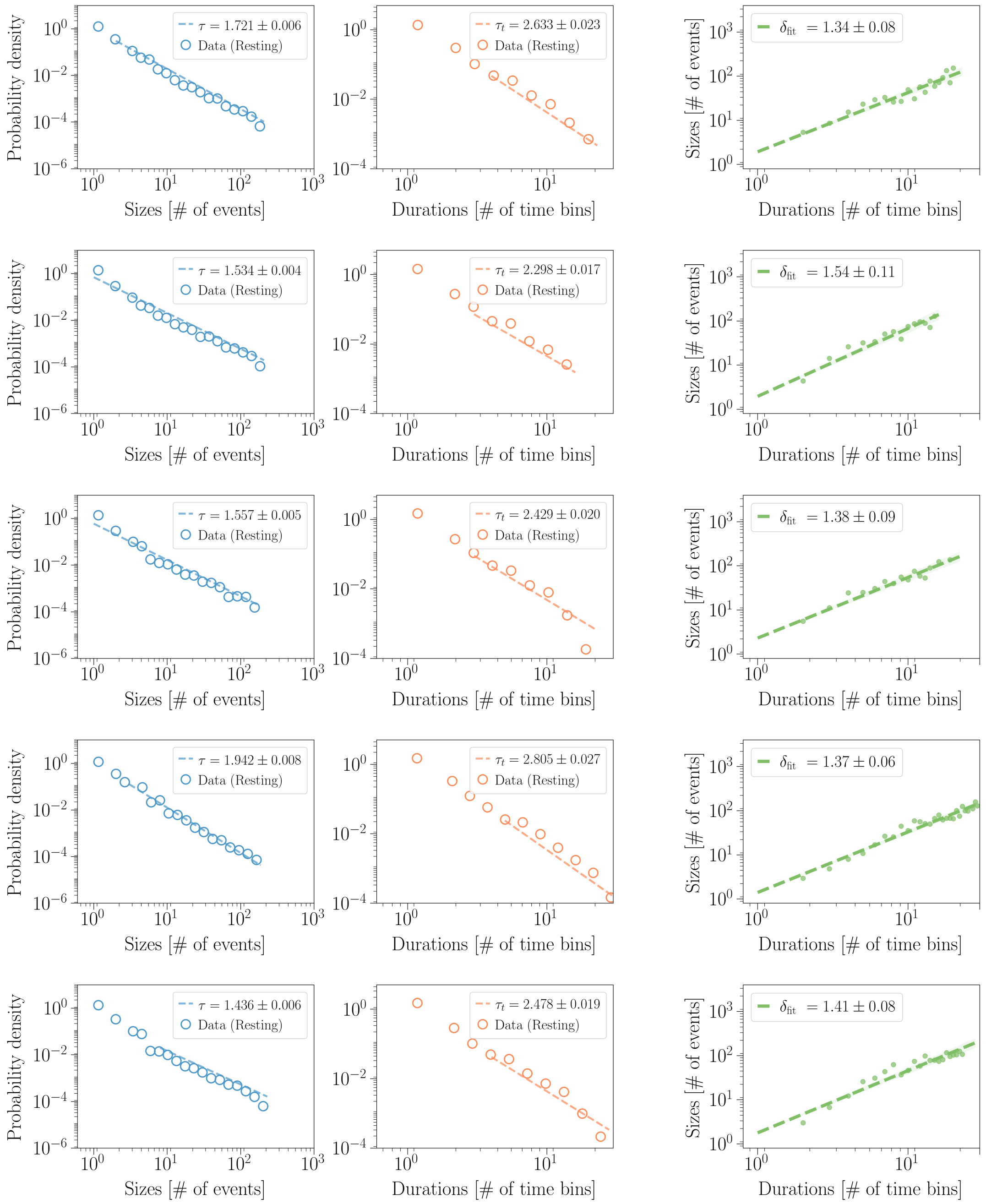

Figure S8: **Avalanches during resting with only negative peaks detected as events.** Distribution of avalanches' sizes (in blue), durations (in red) and average size given duration (in green) in LFPs data obtained considering only the negative peaks as events at resting. We note that the main conclusions that we find as regard LFPs avalanches are also valid when considering only negative peaks: avalanche sizes distributions result power law distributed (all p-values > 0.1) and exponents present some slight changes, due to the exclusions of some events of activity that are visible as positive peaks. See Table S3 for exponents values and p-values. In some rats the exponent τ is closer to the exponent 1.5 typically reported in the literature for nLFPs based avalanches [4, 5]. As it is known from the literature, when considering only negative peaks a hard cutoff is expected in avalanches' sizes, which affects consequently avalanches' durations distribution in a dramatic way - the fitted exponent of avalanche duration is highly unreliable due to the very small range of fitting, as it is possible to see especially in Figure S9. As a consequence we are not able to study the crackling noise relation, since it is highly dependent on the fitting range of τ_t, and we only report the value of $\delta_{\rm fit}$.

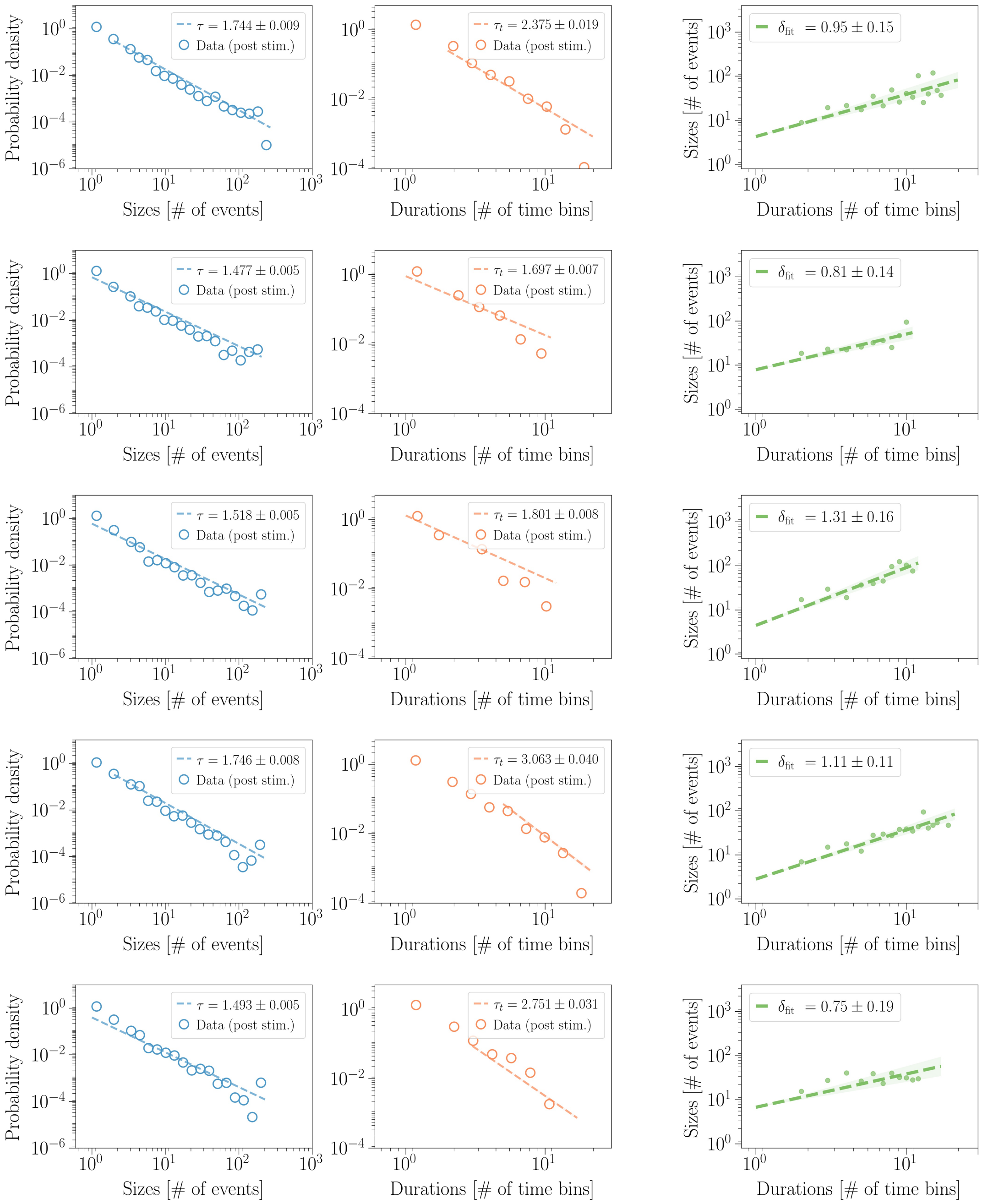

Figure S9: **Avalanches post stimulus with only negative peaks detected as events.** Distribution of avalanches sizes (in blue), durations (in red) and average size given duration (in green) in LFPs data obtained considering only the negative peaks as events post stimulus. We note that bumps in avalanche sizes' distributions are visible even in this case. Avalanches' sizes distributions result power law distributed (all p-values > 0.1, see Table S4).

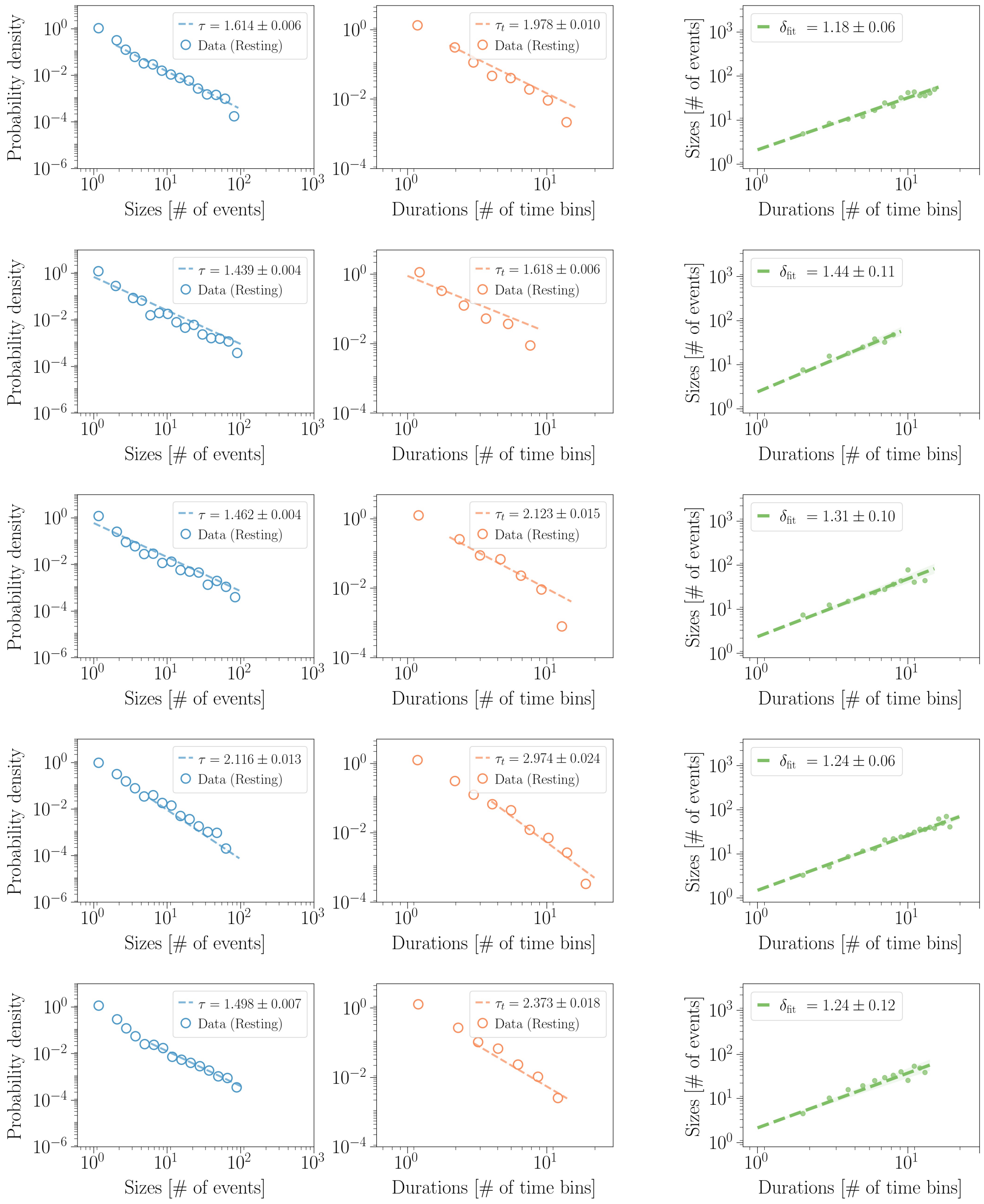

Figure S10: **Avalanches during resting in the superficial layers.** We are able to study LFPs avalanches' distributions considering the superficial layers (layers I-II-II-IV) separately from the bottom layers (layer Va-Vb-VI). In order to enable comparisons with previous works on superficial layers [5], in this analysis we have only considered negative peaks as events. Distribution of avalanches sizes (in blue), durations (in red) and average size given duration (in green). Avalanche sizes distributions are power law distributed (all p-values > 0.1). The exponents of avalanches' sizes are very close to the ones of Fig. S8, that considers all the layers. See also Tables S3 and S5 for exponents values and p-values. Avalanches' durations have a very narrow range as already stressed in Figures S8 and S9.

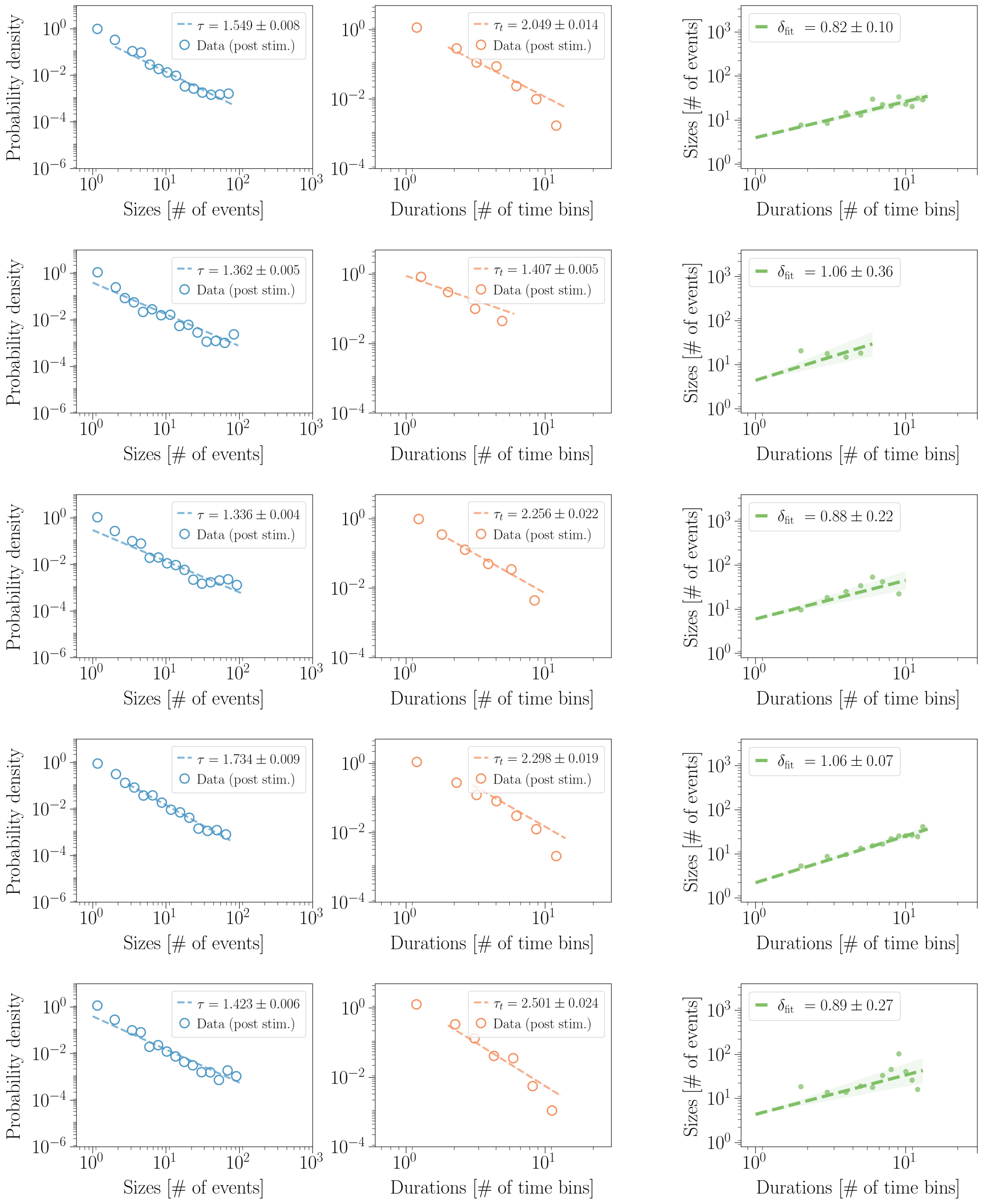

Figure S11: **Avalanches post stimulus in the superficial layers.** Avalanche sizes distributions result power law distributed (all p-values > 0.1, see Table S6 for exponents' values and p-values).

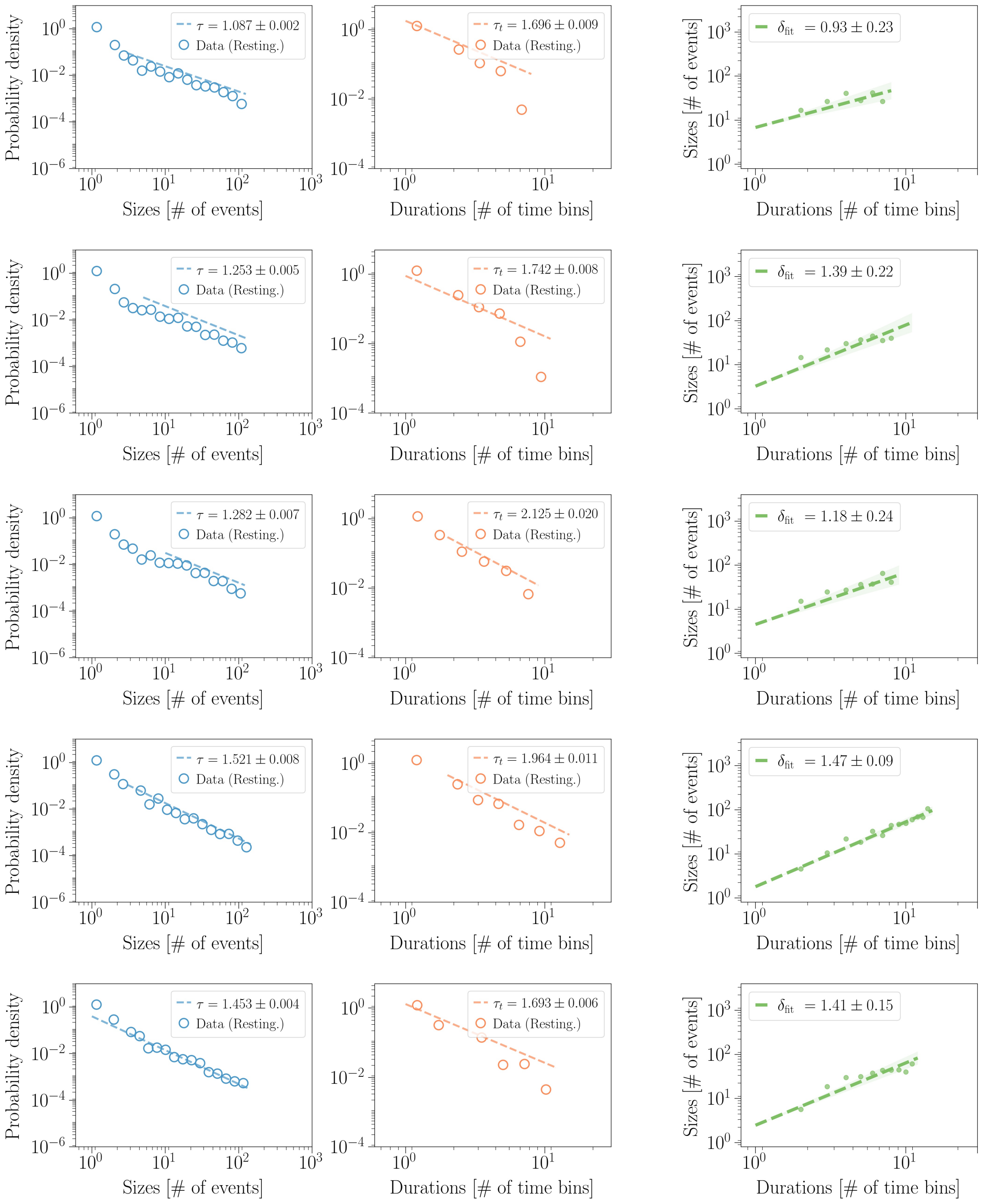

Figure S12: **Avalanches during resting in the bottom layers.** We are able to study LFPs avalanches' distributions considering the superficial layers (layers I-II-II-IV) separately from the bottom layers (layer Va-Vb-VI). Distribution of avalanches sizes (in blue), durations (in red) and average size given duration (in green). Notably, we also find power law distributions for avalanches sizes of the bottom layers (all p-values > 0.1), and differently from other studies [6]. These studies, however, refer to the motor cortex, which has a different neurophysiological function, neuronal composition and network architecture with respect to the somatosensory cortex. Avalanches' sizes exponents are smaller than the ones of the superficial layers (see Tables S5 and S7 for exponents' values and p-values). Avalanches' durations have a very narrow range as already stressed in Figures S8 and S9.

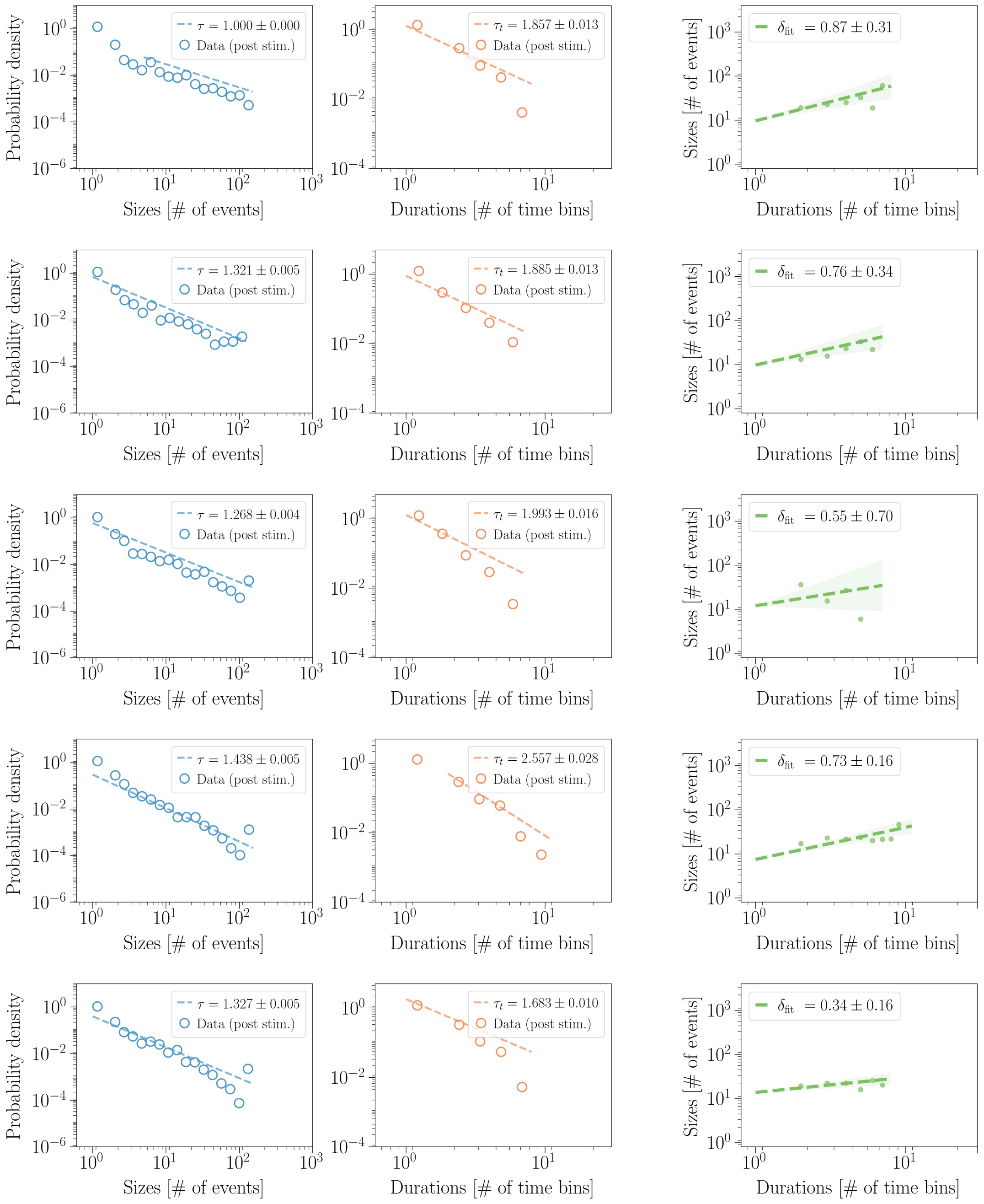

Figure S13: **Avalanches post stimulus in the bottom layers.** Avalanche sizes distributions result power law distributed (all p-values > 0.1, see Table S8 for exponents' values and p-values)

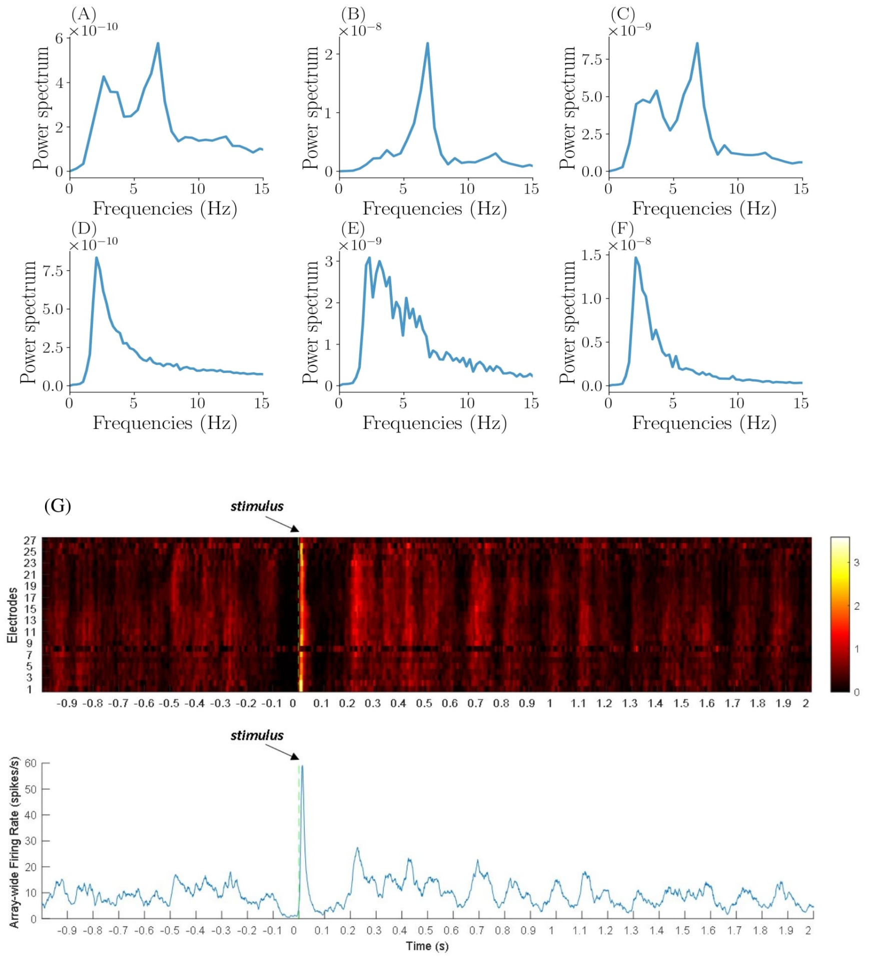

Figure S14: **Differences between evoked response and spontaneous activity.** Here we characterize neural activity in terms of the power spectrum in the case of the LFPs and firing rate in the case of MUAs. A)-B)-C) Power spectrum in the periods after stimuli (up to 2s after the stimulation). A) refers to the average of the power spectrum of the channels belonging to layers I-II, B) to channels in layers III-IV and C) to channels in layers Va-Vb and VI. A dominant peak near 6-7 Hz characterizes the evoked response, whose power is particularly high in the central layers. D)-E)-F) refer to the same subdivision of channels, during spontaneous activity, which is not anymore characterized by the peak at 6 Hz. A peak near 3 Hz is instead present, that can be linked to the effect of the anesthesia. In G) it is possible to appreciate the firing rate of MUAs (top panel: firing rate of each channel, bottom panel: cumulative firing rate across channels), before the stimulation, when the stimulus arrives and after the stimulation.

2 SUPPLEMENTARY TABLES

	# > x_{min} (sizes post stim.)	# > x_{min} (sizes resting.)	# > x_{min} (durations post stim.)	# > x_{min} (durations resting)
Rat 1	342	331	200	200
Rat 2	434	541	579	541
Rat 3	450	175	211	572
Rat 4	409	609	129	118
Rat 5	121	77	134	195

Table S1. Here we report the final number of avalanches after subsampling used to fit the data and obtain the exponents of LFPs avalanches of the main text. In particular here we report the # of sizes (and durations) after subsampling of the time series that fell beyond the x_{min} selected by the fit.

	# > x_{min} (sizes post stim.)	# > x_{min} (sizes resting.)	# > x_{min} (durations post stim.)	# > x_{min} (durations resting)
Rat 1	295	963	253	1164
Rat 2	188	1210	338	2233
Rat 3	130	919	205	139
Rat 4	771	482	397	1160

Table S2. Here we report the final number of avalanches after subsampling used to fit the data and obtain the exponents of MUAs avalanches of the main text. In particular here we report the # of sizes (or durations) after subsampling of the time series that fell beyond the x_{min} selected by the fit.

	Rat 1	Rat 2	Rat 3	Rat 4	Rat 5
τ	1.721 ± 0.006	1.534 ± 0.004	1.557 ± 0.005	1.942 ± 0.008	1.436 ± 0.006
p-values	0.62	0.64	0.66	0.62	0.81
# of samples > x_{min}	771	958	625	719	273

Table S3. Exponents, p-values, and number of samples (obtained after the sub-sampling procedure) of LFPs avalanches' sizes considering only negative peaks as events in resting state periods (Figure S8).

	Rat 1	Rat 2	Rat 3	Rat 4	Rat 5
τ	1.743 ± 0.009	1.477 ± 0.005	1.518 ± 0.005	1.746 ± 0.008	1.493 ± 0.005
p-values	0.48	0.66	0.48	0.32	0.40
# of samples > x_{min}	316	468	531	411	566

Table S4. Exponents, p-values, and number of samples (obtained after the sub-sampling procedure) of LFPs avalanches' sizes considering only negative peaks as events in post stimuli periods (Figure S9).

	Rat 1	Rat 2	Rat 3	Rat 4	Rat 5
τ	1.614 ± 0.006	1.439 ± 0.004	1.461 ± 0.004	2.12 ± 0.012	1.498 ± 0.007
p-values	0.94	0.94	0.92	0.86	0.88
$\#$ of samples $> x_{min}$	574	592	584	369	245

Table S5. Exponents, p-values, and number of samples (obtained after the sub-sampling procedure) of LFPs avalanches' sizes in the superficial layers (considering only negative peaks as events) in resting state periods (Figure S10).

	Rat 1	Rat 2	Rat 3	Rat 4	Rat 5
τ	1.549 ± 0.008	1.36 ± 0.005	1.336 ± 0.004	1.734 ± 0.009	1.423 ± 0.006
p-values	0.67	0.40	0.36	0.67	0.57
$\#$ of samples $> x_{min}$	236	297	316	332	253

Table S6. Exponents, p-values, and number of samples (obtained after the sub-sampling procedure) of LFPs avalanches' sizes in the superficial layers (considering only negative peaks as events) in post stimuli periods Figure S11).

	Rat 1	Rat 2	Rat 3	Rat 4	Rat 5
τ	1.086 ± 0.002	1.253 ± 0.0045	1.281 ± 0.007	1.521 ± 0.007	1.453 ± 0.004
p-values	0.87	0.91	0.87	0.86	0.69
$\#$ of samples $> x_{min}$	152	166	95	233	654

Table S7. Exponents, p-values, and number of samples (obtained after the sub-sampling procedure) of LFPs avalanches' sizes in the bottom layers (considering only negative peaks as events) in resting state periods Figure S12).

	Rat 1	Rat 2	Rat 3	Rat 4	Rat 5
τ	1.000 ± 0.001	1.321 ± 0.005	1.268 ± 0.004	1.438 ± 0.005	1.327 ± 0.005
p-values	0.93	0.39	0.45	0.54	0.30
$\#$ of samples $> x_{min}$	90	221	190	335	243

Table S8. Exponents, p-values, and number of samples (obtained after the sub-sampling procedure) of LFPs avalanches' sizes in the bottom layers (considering only negative peaks as events) in post stimuli periods Figure S13).

	post. stim.	resting
Rat 1	$0.56 \pm 0..01$	0.45 ± 0.01
Rat 2	0.44 ± 0.02	0.30 ± 0.01
Rat 3	0.65 ± 0.01	0.48 ± 0.02
Rat 4	0.62 ± 0.01	0.40 ± 0.01
Rat 5	0.58 ± 0.01	0.46 ± 0.01

Table S9. Here we report the Kuramoto order parameters measured in our LFPs data in the 200ms after stimuli and in periods of resting state. The Kuramoto order parameter is defined as $\langle | \sum_{i=1}^{N} e^{i\phi_i(t)}/N | \rangle_t$, were the average is performed in the time interval considered and N is the number of channels. $\phi_i(t)$ is the instantaneous phase of channel i, that is extracted from the real-valued signal $x_i(t)$ by taking the phase of its analytic representation, that is $x_i(t) + i\mathcal{H}(t)$, with $\mathcal{H}_i(t)$ the Hilbert transform of the signal. It is possible to see that the Kuramoto parameter is higher is the periods after stimuli with respect to the resting state periods.

REFERENCES

[1]R. Quian Quiroga, Z. Nadasdy, and Y. Ben-Shaul. Unsupervised Spike Detection and Sorting with Wavelets and Superparamagnetic Clustering. *Neural Computation*, 16(8):1661–1687, 08 2004.

[2]W. Shew, W. Clawson, J. Pobst, and al. Adaptation to sensory input tunes visual cortex to criticality. *Nature Phys.*, 11:659–663, 2015.

[3]Martin Gerlach and Eduardo G. Altmann. Testing statistical laws in complex systems. *Phys. Rev. Lett.*, 122:168301, 2019.

[4]John M. Beggs and Dietmar Plenz. Neuronal avalanches in neocortical circuits. *Journal of Neuroscience*, 12:23(35):11167–11177, 2003.

[5]E. D. Gireesh and D. Plenz. Neuronal avalanches organize as nested theta- and beta/gamma-oscillations during development of cortical layer 2/3. *Proc Natl Acad Sci U S A*, page 7576-7581, 2008.

[6]Zhengyu Ma, Haixin Liu, Takaki Komiyama, and Ralf Wessel. Stability of motor cortex network states during learning-associated neural reorganizations. *Journal of Neurophysiology*, 124(5):1327–1342, 2020. PMID: 32937084.
